# A stable numerical method for the dynamics of fluidic membranes

**DOI:** 10.1007/s00211-015-0787-5

**Published:** 2016-02-23

**Authors:** John W. Barrett, Harald Garcke, Robert Nürnberg

**Affiliations:** 1 Department of Mathematics, Imperial College London, London, SW7 2AZ UK; 2 Fakultät für Mathematik, Universität Regensburg, 93040 Regensburg, Germany

**Keywords:** 65M60, 65M12, 76M10, 76Z99, 92C05, 35Q35, 76D05

## Abstract

We develop a finite element scheme to approximate the dynamics of two and three dimensional fluidic membranes in Navier–Stokes flow. Local inextensibility of the membrane is ensured by solving a tangential Navier–Stokes equation, taking surface viscosity effects of Boussinesq–Scriven type into account. In our approach the bulk and surface degrees of freedom are discretized independently, which leads to an unfitted finite element approximation of the underlying free boundary problem. Bending elastic forces resulting from an elastic membrane energy are discretized using an approximation introduced by Dziuk (Numer Math 111:55-80, 2008). The obtained numerical scheme can be shown to be stable and to have good mesh properties. Finally, the evolution of membrane shapes is studied numerically in different flow situations in two and three space dimensions. The numerical results demonstrate the robustness of the method, and it is observed that the conservation properties are fulfilled to a high precision.

## Introduction

The evolution of lipid bilayer membranes is driven by the bending energy, which involves the curvature of the membrane, and hydrodynamics. Lipid membranes typically form vesicles, i.e. bag-like structures containing fluid, which are surrounded by a possibly different fluid. The omnipresence of membranes in biological systems has led to a growing interest in vesicles over the past decades. Much of the work on vesicles was motivated by the fact that their shape at rest resembles the biconcave forms of red blood cells. It is the goal of this paper to introduce, and analyze, a finite element method of a model for the evolution of lipid membranes, which was introduced by Arroyo and DeSimone [2].

Their model couples a tangential Navier–Stokes system on the membrane to a bulk Navier–Stokes system. On the membrane, forces stemming from a first variation of the curvature energy appear. In this paper, we introduce a finite element method discretizing the bulk and surface degrees of freedoms independently, which leads to an unfitted approximation of the two-phase flow problem. The forces resulting from the elastic membrane energy are discretized using an approach that was introduced by Dziuk [18] for Willmore flow. The two Navier–Stokes systems are coupled and subsequently discretized with the help of a suitable variational formulation, which allows us to show a stability bound for the discretization of the underlying complex free boundary problem. It will turn out that the surface finite element mesh has good mesh properties also for strongly deforming membrane evolutions. This fact results from a suitable discrete local incompressibility condition on the surface, which we will also analyse. Before we state the governing equations and the numerical method in more detail, we discuss the physical background and review approaches used by other authors to numerically solve similar problems.

The bending energy for a lipid membrane used in this paper is

$$\begin{aligned} E_\alpha ({\varGamma }) = \alpha \,E({\varGamma })\,, \quad \text {with}\quad E({\varGamma }) = \tfrac{1}{2} \int _{{\varGamma }} \varkappa ^2 \;\mathrm{d}{\mathscr {H}}^{d-1}, \end{aligned}$$

where the bilayer is modelled as a closed hypersurface ${\varGamma }$ in $\mathbb {R}^d$, $d=2$ or 3. By $\varkappa$ we denote the mean curvature (the sum of the principal curvatures) of ${\varGamma }$, $\alpha \in {\mathbb R}_{>0}$ is the bending rigidity and $\;\mathrm{d}{\mathscr {H}}^{d-1}$ indicates integration with respect to the $(d-1)$-dimensional surface measure. In the simplest energetical model for vesicles one minimizes the energy $E_\alpha ({\varGamma })$ under the constraints that the area of ${\varGamma }$ is fixed and that ${\varGamma }$ encloses a fixed volume. The latter is due to the fact that the osmotic conditions of the fluids surrounding the membrane lead to a fixed volume. Furthermore, the vesicle can be considered as locally incompressible, which leads to a fixed total surface area. For a deeper physical discussion of these conditions we refer to the overview article [39], where also other aspects of fluidic membranes and vesicles are thoroughly discussed.

In the fluid regions, ${\varOmega }_-$ and ${\varOmega }_+$, inside and outside of the membrane, one requires the incompressible Navier–Stokes equations, i.e.At typical temperatures the membrane itself is in a fluidic state, which leads to the fact that on the membrane the incompressible surface Navier–Stokes equationshave to hold. Here $\partial _t^\bullet$ denotes the material time derivative on ${\varGamma }$,  is the surface stress tensor, ${\vec {\nu }}$ is the unit normal on ${\varGamma }$ and  describes stresses acting on the membrane via the normal stresses  from both sides of the membrane (see Sect. 2 for precise definitions). The operator $\nabla _{\mathrm{s}}\,.$ is the surface divergence and $\nabla _{\mathrm{s}}\,.\,{\vec {u}} = 0$ models the fact that the membrane is locally incompressible.

Interfacial fluid mechanics was first thoroughly discussed by Scriven [38], generalizing earlier ideas of Boussinesq. In this context the surface stress tensor  was first introduced, and is hence called the Boussinesq–Scriven tensor. In addition, $\alpha \, f_{\varGamma }\,{\vec {\nu }}$ models forces acting on the membrane, which result from the curvature elasticity $E_\alpha ({\varGamma })$. The forces act in a direction normal to the membrane and $f_{\varGamma }$ is given as minus the first variation of $E({\varGamma })$, i.e.

1.1 $$\begin{aligned} f_{\varGamma }= -{\varDelta }_\mathrm{s}\,\varkappa -\varkappa \,|\nabla _{\mathrm{s}}\,{\vec {\nu }}|^2 +\tfrac{1}{2}\,\varkappa ^3, \end{aligned}$$

where ${\varDelta }_\mathrm{s}$ is the surface Laplacian and $\nabla _{\mathrm{s}}$ is the surface gradient.

In recent years, many papers have appeared which numerically approximate the $L^2$-gradient flow equation related to the Willmore energy $E({\varGamma })$, i.e.

1.2 $$\begin{aligned} \mathscr {V} = -{\varDelta }_\mathrm{s}\,\varkappa -\varkappa \,|\nabla _{\mathrm{s}}\,{\vec {\nu }}|^2 +\tfrac{1}{2}\,\varkappa ^3, \end{aligned}$$

where $\mathscr {V}$ is the normal velocity of the evolving membrane ${\varGamma }$. This geometric evolution equation is called Willmore flow, and we refer to [6, 7, 14, 15, 17, 18, 22, 29] for different computational approaches to Willmore flow. Since the enclosed volume and the total surface area are preserved for lipid membranes, the volume and area preserving variant of (1.2), which is called Helfrich flow, is of particular interest. Helfrich flow has been considered numerically in e.g. [6, 12]. Other authors included additional physical effects in the geometrical model, such as lateral inhomogeneity and line tension effects, see [20, 31]. In [13] a fluid-membrane system, in which forces resulting from the Willmore energy act on an interior flow, is considered. In that model surface area is maintained with the help of a global Lagrange multiplier.

As pointed out above, the membrane is locally incompressible and hence the condition $\nabla _{\mathrm{s}}\,.\,{\vec {u}} = 0$ should be enforced on the flow. This condition has been dealt with in numerical simulations in [27, 35, 36] within a level set context, in [1, 24] with the help of a phase field approach and in [23] by using an immersed boundary method. In these approaches the local incompressibility constraint on the membrane is enforced by a Lagrange multiplier leading to an inhomogeneous surface pressure. However, in the computations in the latter paper the constraint is relaxed by a spring-like elastic force. In addition, there exists work on the surface Stokes system without taking the bulk fluid flow into account. There the volume conservation is enforced by a global Lagrange multiplier. We refer to [33, 34] for numerical results using this modelling variant. The only numerical work taking simultaneously surface and bulk viscosity effects in the fluidic membrane evolution into account is [3]. However, these results are restricted to the axisymmetric situation. In addition, their numerical method cannot be shown to be stable, as is the case for all of the above numerical methods.

We also refer to numerical work in [21, 26, 30, 40] on the evolution of red blood cells, which study the influence of the elastic effects resulting from the cytoskeleton on the membrane evolution. Finally, we mention that analytical well-posedness issues for the model considered in this paper are currently being addressed in [25, 28].

Building on earlier work by the present authors on two-phase flow and by Dziuk [18] on Willmore flow, it is the main goal of this paper to introduce and analyze a numerical method for the full membrane evolution problem. Our numerical approach has the following features:

- The bulk and surface degrees of freedom are discretized with standard bulk and surface finite elements leading to an unfitted finite element method.
- The effects of the bulk fluid and of the fluidic membrane are taken into account simultaneously. In particular, surface viscous effects are accounted for through the Boussinesq–Scriven law.
- Local volume and local membrane area conservation result naturally from the volume and surface incompressibility conditions. Local area conservation can be shown for a continuous-in-time semidiscrete variant of our proposed scheme. In addition, for a simple modification of our scheme, which can be interpreted as a virtual element method, see e.g. [41], volume conservation properties can also be shown.
- Elastic forcing from the curvature energy $E({\varGamma })$ is taken into account, and this is discretized with the help of a weak formulation due to [18].
- Stability of a semidiscrete version can be shown. To our knowledge, this is the first stability result in the literature for a numerical approximation of the dynamics of fluidic membranes.
- The interface is advected with the help of the fluid velocity. In other fluid flow problems with a free boundary this typically leads to distortions of the parametric surface mesh, see the discussion in [4]. However, in our case the local surface area conservation $\nabla _{\mathrm{s}}\,.\,{\vec {u}} = 0$ guarantees that the surface mesh quality remains good during the evolution, see Remark 4.1 and the numerical simulations in Sect. 7. We also refer to [32], where a strategy for the tangential redistribution of mesh points by conserving the relative surface area during the evolution was designed.
- Fully three dimensional simulations, without making any symmetry assumptions, have been performed, and to the knowledge of the authors this paper presents the first such numerical computations for the full fluidic membrane problem, i.e. taking the bulk viscosity, the surface viscosity and the local incompressibility of the bulk and surface fluid into account.

The outline of the paper is as follows. After introducing the governing equations in Sect. 2, we present a weak formulation in Sect. 3. This weak formulation is the basis for our semidiscrete and fully discrete finite element approximations, which are formulated and analyzed in Sects. 4 and 5. After stating the solutions methods in Sect. 6, we present numerical simulations in Sect. 7.

## Governing equations

**Fig. 1:**
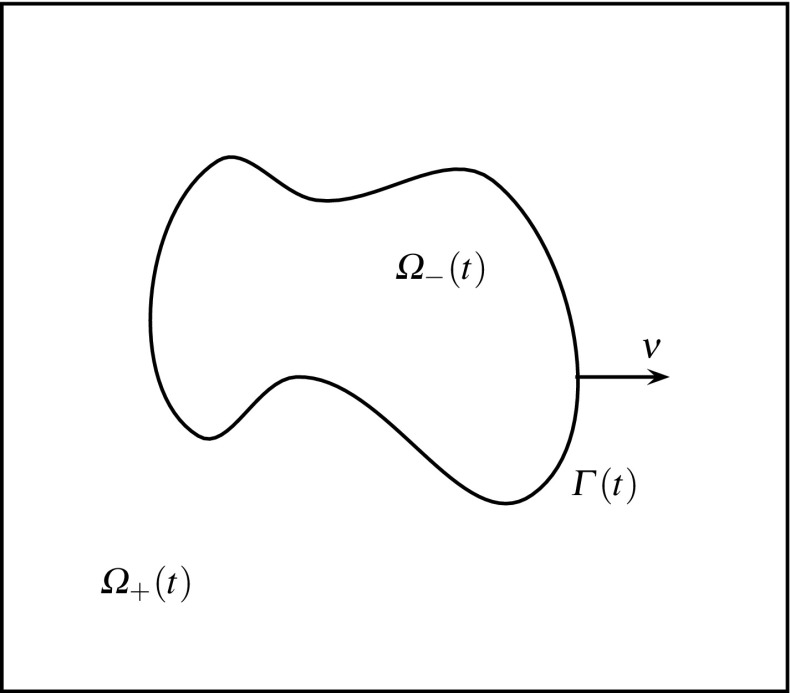
The domain ${\varOmega }$ in the case $d=2$

In this section we state the equations governing the evolution of fluidic membranes, as introduced in [2]. Let ${\varOmega }\subset \mathbb {R}^d$ be a given domain, where $d=2$ or $d=3$. We seek a time dependent interface $({\varGamma }(t))_{t\in [0,T]}$, ${\varGamma }(t)\subset {\varOmega }$, which for all $t\in [0,T]$ separates ${\varOmega }$ into a domain ${\varOmega }_+(t)$, occupied by the outer phase, and a domain ${\varOmega }_{-}(t):={\varOmega }\setminus \overline{{\varOmega }}_+(t)$, which is occupied by the inner phase, see Fig. 1 for an illustration. For later use, we assume that $({\varGamma }(t))_{t\in [0,T]}$ is a sufficiently smooth evolving hypersurface without boundary that is parameterized by ${\vec {x}}(\cdot ,t):{\varUpsilon }\rightarrow {\mathbb R}^d$, where ${\varUpsilon }\subset {\mathbb R}^d$ is a given reference manifold, i.e. ${\varGamma }(t) = {\vec {x}}({\varUpsilon },t)$. Then

2.1 $$\begin{aligned} {\vec {\mathscr {V}}}({\vec {z}}, t) := {\vec {x}}_t({\vec {q}}, t) \qquad \forall \ {\vec {z}} = {\vec {x}}({\vec {q}},t) \in {\varGamma }(t) \end{aligned}$$

defines the velocity of ${\varGamma }(t)$, and $\mathscr {V} := {\vec {\mathscr {V}}} \,.\,{\vec {\nu }}$ is the normal velocity of the evolving hypersurface ${\varGamma }(t)$, where ${\vec {\nu }}(t)$ is the unit normal on ${\varGamma }(t)$ pointing into ${\varOmega }_+(t)$. Moreover, we define the space-time surface ${\mathscr {G}_T}:= \bigcup _{t \in [0,T]} {\varGamma }(t) \times \{t\}$.

Let $\rho (t) = \rho _+\,\mathrm {\mathscr {X}}_{{\varOmega }_+(t)} + \rho _-\,\mathrm {\mathscr {X}}_{{\varOmega }_-(t)}$, with $\rho _\pm \in {\mathbb R}_{\ge 0}$, denote the fluid densities, where here and throughout $\mathrm {\mathscr {X}}_{\mathscr {A}}$ defines the characteristic function for a set $\mathscr {A}$. Denoting by ${\vec {u}} : {\varOmega }\times [0, T] \rightarrow {\mathbb R}^d$ the fluid velocity, by  the stress tensor, and by ${\vec {f}} : {\varOmega }\times [0, T] \rightarrow {\mathbb R}^d$ a possible volume force, the incompressible Navier–Stokes equations in the two phases are given by 2.2a
2.2b
2.2c
2.2d where $\partial {\varOmega }=\partial _1{\varOmega }\cup \partial _2{\varOmega }$, with $\partial _1{\varOmega }\cap \partial _2{\varOmega }=\emptyset$, denotes the boundary of ${\varOmega }$ with outer unit normal ${\vec {n}}$. Hence (2.2c) prescribes a possibly inhomogeneous Dirichlet condition for the velocity on $\partial _1{\varOmega }$, which collapses to the standard no-slip condition when ${\vec {g}} = {\vec {0}}$, while (2.2d) prescribes a stress-free condition on $\partial _2{\varOmega }$. Throughout this paper we assume that $\mathscr {H}^{d-1}(\partial _1{\varOmega }) > 0$. We will also assume w.l.o.g. that ${\vec {g}}$ is extended so that ${\vec {g}} : {\varOmega }\rightarrow {\mathbb R}^d$. In addition, the stress tensor in (2.2a) is defined by2.3where  denotes the identity matrix and  is the rate-of-deformation tensor, with $\nabla \,{\vec {u}} = \left( \partial _{x_j}\,u_i \right) _{i,j=1}^d$. Moreover, $p : {\varOmega }\times [0, T] \rightarrow {\mathbb R}$ is the pressure and $\mu (t) = \mu _+\,\mathrm {\mathscr {X}}_{{\varOmega }_+(t)} + \mu _-\,\mathrm {\mathscr {X}}_{{\varOmega }_-(t)}$, with $\mu _\pm \in {\mathbb R}_{>0}$, denotes the dynamic viscosities in the two phases. On the free surface ${\varGamma }(t)$, the following conditions need to hold: 2.4a
2.4b
2.4c
2.4d where $\rho _{\varGamma }\in {\mathbb R}_{\ge 0}$ denotes the surface material density, $\alpha \in {\mathbb R}_{>0}$ is the bending rigidity and ${\vec {f}}_{\varGamma }:= f_{\varGamma }\,{\vec {\nu }}$ is defined by (1.1). In addition, $\nabla _{\mathrm{s}}\,.\,$ denotes the surface divergence on ${\varGamma }(t)$, and the surface stress tensor is given by2.5where $\mu _{\varGamma }\in {\mathbb R}_{\ge 0}$ is the interfacial shear viscosity and $p_{\varGamma }$ denotes the surface pressure, which acts as a Lagrange multiplier for the incompressibility condition (2.4c). Here 2.6ais the projection onto the tangent space of ${\varGamma }(t)$, and2.6b is the surface rate-of-deformation tensor. Here  denotes the surface gradient on ${\varGamma }(t)$, and $\nabla _{\mathrm{s}}\,{\vec {u}} = \left( \partial _{s_j}\, u_i \right) _{i,j=1}^d$. Moreover, as usual, $[{\vec {u}}]_-^+ := {\vec {u}}_+ - {\vec {u}}_-$ and  denote the jumps in velocity and normal stress across the interface ${\varGamma }(t)$. Here and throughout, we employ the shorthand notation ${\vec {b}}_\pm := {\vec {b}}\mid _{{\varOmega }_\pm (t)}$ for a function ${\vec {b}} : {\varOmega }\times [0,T] \rightarrow {\mathbb R}^d$; and similarly for scalar and matrix-valued functions. In addition,

2.7 $$\begin{aligned} \partial _t^\bullet \, \zeta = \zeta _t + {\vec {u}} \,.\,\nabla \,\zeta \qquad \forall \ \zeta \in H^1({\mathscr {G}_T}) \end{aligned}$$

denotes the material time derivative of $\zeta$ on ${\varGamma }(t)$. We compute $\partial _t^\bullet \, \zeta$ with the help of an extension of $\zeta$ to a neighborhood of ${\mathscr {G}_T}$. Here we stress that the derivative in (2.7) is well-defined, and depends only on the values of $\zeta$ on ${\mathscr {G}_T}$, even though $\zeta _t$ and $\nabla \,\zeta$ do not make sense separately for a function defined on ${\mathscr {G}_T}$; see e.g. [19, p. 324]. The system (2.2a–d), (2.3), (2.4a–d), (2.5) is closed with the initial conditions

2.8 $$\begin{aligned} {\varGamma }(0) = {\varGamma }_0 \,, \quad \rho \,{\vec {u}}(\cdot ,0) = \rho \,{\vec {u}}_0 \quad \text{ in } {\varOmega }\,, \quad \rho _{\varGamma }\,{\vec {u}}(\cdot ,0) = \rho _{\varGamma }\,{\vec {u}}_0 \quad \text{ on } {\varGamma }_0, \end{aligned}$$

where ${\varGamma }_0 \subset {\varOmega }$ and ${\vec {u}}_0 : {\varOmega }\rightarrow {\mathbb R}^d$ are given initial data satisfying $\rho \,\nabla \,.\,{\vec {u}}_0 = 0$ in ${\varOmega }$, $\rho _{\varGamma }\,\nabla _{\mathrm{s}}\,.\,{\vec {u}}_0 = 0$ on ${\varGamma }_0$ and $\rho _+\,{\vec {u}}_0 = \rho _+\,{\vec {g}}$ on $\partial _1{\varOmega }$. Of course, in the case $\rho _- = \rho _+ = \rho _{\varGamma }= 0$ the initial data ${\vec {u}}_0$ is not needed. Similarly, in the case $\rho _- = \rho _+ = 0$ and $\rho _{\varGamma }> 0$ the initial data ${\vec {u}}_0$ is only needed on ${\varGamma }_0$. However, for ease of exposition, and in view of the unfitted nature of our numerical method, we will always assume that ${\vec {u}}_0$, if required, is given on all of ${\varOmega }$.

It is not difficult to show that the conditions (2.2b) enforce volume preservation for the phases, while (2.4c) leads to the conservation of the total surface area $\mathscr {H}^{d-1}({\varGamma }(t))$, see Sect. 3 below for the relevant proofs. As an immediate consequence we obtain that spheres remain spheres, and that spheres with a zero bulk velocity are stationary solutions. In addition, in the case $d=2$ the condition (2.4c) immediately implies that  on ${\varGamma }(t)$, which means that in two space dimensions the problem is independent of the value of $\mu _{\varGamma }$.

Furthermore, we note that2.9Here $\varkappa$ denotes the mean curvature of ${\varGamma }(t)$, i.e. the sum of the principal curvatures $\varkappa _i$, $i=1,\ldots ,d-1$, of ${\varGamma }(t)$, where we have adopted the sign convention that $\varkappa$ is negative where ${\varOmega }_-(t)$ is locally convex. In particular, it holds that

2.10 $$\begin{aligned} {\varDelta }_\mathrm{s}\, {\vec {\mathrm{id}}} = \varkappa \, {\vec {\nu }} =: {\vec {\varkappa }} \qquad \text{ on } {\varGamma }(t), \end{aligned}$$

where ${\varDelta }_\mathrm{s} = \nabla _{\mathrm{s}}\,.\,\nabla _{\mathrm{s}}$ is the Laplace–Beltrami operator on ${\varGamma }(t)$.

Assuming there are two solutions $\{({\varGamma }(t), {\vec {u}}(\cdot ,t), p^{(i)}(\cdot ,t), p_{\varGamma }^{(i)}(\cdot ,t))\}_{t \in [0,T]}$, $i=1,2$, to the problem (2.2a–d), (2.3), (2.4a–d), (2.5) and (2.8), then it follows from (2.3) and (2.9) that

2.11a $$\begin{aligned} \nabla \,\bar{p}_\pm&= {\vec {0}}&\qquad \text {in}\ {\varOmega }_\pm (t), \end{aligned}$$

2.11b $$\begin{aligned} \nabla _{\mathrm{s}}\,\bar{p}_{\varGamma }+ \varkappa \,\bar{p}_{\varGamma }\,{\vec {\nu }}&= [\bar{p}\,{\vec {\nu }}]^+_-&\qquad \text {on}\ {\varGamma }(t), \end{aligned}$$

where $\bar{p} = p^{(1)} - p^{(2)}$ and $\bar{p}_{\varGamma }= p_{\varGamma }^{(1)} - p_{\varGamma }^{(2)}$. Therefore $\bar{p}_\pm$ is constant on ${\varOmega }_\pm (t)$. In addition, since $\nabla _{\mathrm{s}}\,\bar{p}_{\varGamma }$ is tangential, we obtain that $\nabla _{\mathrm{s}}\,\bar{p}_{\varGamma }={\vec {0}}$, and hence $\bar{p}_{\varGamma }$ is a constant. Moreover, (2.11b) implies that $\varkappa \,\bar{p}_{\varGamma }= \bar{p}_+ - \bar{p}_-$. So if $\varkappa$ is not constant, which is the case if ${\varGamma }(t)$ is not a sphere, then $\bar{p}_{\varGamma }= 0$ and $\bar{p}_+ = \bar{p}_-$. Hence $p_{\varGamma }$ in this case is unique, and *p* is unique in ${\varOmega }$ up to an additive constant. If $\varkappa$ is constant, however, i.e. if ${\varGamma }(t)$ is a sphere, then $p_{\varGamma }$ is only unique up to an additive constant, which is fixed by the two additive constants in the bulk phases. For more details see the discussion around (3.13) below.

Finally, we recall that the source term ${\vec {f}}_{\varGamma }= f_{\varGamma }\,{\vec {\nu }}$ in (2.4b), with $f_{\varGamma }$ defined in (1.1), is the first variation of $E({\varGamma }(t))$, i.e.

$$\begin{aligned} \tfrac{1}{2}\,\frac{\mathrm{d}}{\mathrm{d}t}\left\langle \varkappa , \varkappa \right\rangle _{{\varGamma }(t)} = - \left\langle f_{\varGamma }, {\mathscr {V}} \right\rangle _{{\varGamma }(t)} = - \left\langle {\vec {f}}_{\varGamma }, {\vec {\mathscr {V}}} \right\rangle _{{\varGamma }(t)}, \end{aligned}$$

where $\langle \cdot , \cdot \rangle _{{\varGamma }(t)}$ denotes the $L^2$–inner product on ${\varGamma }(t)$. It does not appear possible to derive a stable discretization of the system (2.2a–d), (2.3), (2.4a–d), (2.5) based on the formulation (1.1). Hence in this paper we will make use of the stable approximation of Willmore flow introduced in [18], which is based on a discretization of the curvature vector ${\vec {\varkappa }} = \varkappa \,{\vec {\nu }}$, and on the identity2.12where we note that our notation is such that $\nabla _{\mathrm{s}}\,{\vec {\chi }} = (\nabla _{{\varGamma }}\,{\vec {\chi }})^\mathrm{T}$, with $\nabla _{{\varGamma }}\,{\vec {\chi }}=\left( \partial _{s_i}\, \chi _j \right) _{i,j=1}^d$ defined as in [18].

## Weak formulation

Before introducing our finite element approximation, we will state an appropriate weak formulation. With this in mind, we introduce the following function spaces for a given ${\vec {b}} \in [H^1({\varOmega })]^d$:

$$\begin{aligned} \mathbb {U}({\vec {b}})&:= \{ {\vec {\varphi }} \in [H^1({\varOmega })]^d : {\vec {\varphi }} = {\vec {b}} \ \text{ on } \partial _1{\varOmega }\},\\ \mathbb {V}({\vec {b}})&:= L^2(0,T; \mathbb {U}({\vec {b}})) \cap H^1(0,T;[L^2({\varOmega })]^d),\\ \mathbb {V}_{\varGamma }({\vec {b}})&:= \{ {\vec {\varphi }} \in \mathbb {V}({\vec {b}}) : {\vec {\varphi }} \mid _{\mathscr {G}_T}\in [H^1({\mathscr {G}_T})]^d \}. \end{aligned}$$

In addition, we let $\mathbb {P}:= L^2({\varOmega })$ and define

$$\begin{aligned} {\widehat{\mathbb {P}}} := {\left\{ \begin{array}{ll} \{\eta \in \mathbb {P}: \int _{\varOmega }\eta \;\mathrm{d}{\mathscr {L}}^{d}=0 \} &{} \quad \text {if } \mathscr {H}^{d-1}(\partial _2{\varOmega }) = 0, \\ \mathbb {P}&{} \quad \text {if } \mathscr {H}^{d-1}(\partial _2{\varOmega }) > 0. \end{array}\right. } \end{aligned}$$

Letting $(\cdot ,\cdot )$ and $\langle \cdot ,\cdot \rangle _{\partial _2{\varOmega }}$ denote the $L^2$–inner products on ${\varOmega }$ and $\partial _2{\varOmega }$, respectively, we recall from [11] that it follows from (2.2a–d), (2.4a, d) and (2.3) that

3.1 $$\begin{aligned}&(\rho \,[{\vec {u}}_t + ({\vec {u}} \,.\,\nabla )\,{\vec {u}}], {\vec {\xi }}) \nonumber \\&\qquad = \tfrac{1}{2}\left[ \frac{\mathrm{d}}{\mathrm{d}t}(\rho \,{\vec {u}}, {\vec {\xi }}) + (\rho \,{\vec {u}}_t, {\vec {\xi }}) - ( \rho \,{\vec {u}}, {\vec {\xi }}_t) + (\rho , [({\vec {u}}\,.\,\nabla )\,{\vec {u}}]\,.\,{\vec {\xi }} - [({\vec {u}}\,.\,\nabla )\,{\vec {\xi }}]\,.\,{\vec {u}}) \right] \nonumber \\&\qquad \quad + \tfrac{1}{2}\,\rho _+ \left\langle {\vec {u}} \,.\,{\vec {n}}, {\vec {u}}\,.\,{\vec {\xi }} \right\rangle _{\partial _2{\varOmega }} \qquad \forall \ {\vec {\xi }} \in \mathbb {V}({\vec {0}}) \end{aligned}$$

and3.2where we have also noted for symmetric matrices  that  for all . Only slip or free-slip conditions were considered in [11], and so the boundary integral over $\partial _2{\varOmega }$ did not appear there. But it is easily established that the more general (3.1) also holds, on noting [11, (3.2)].

We define, similarly to (2.7), the following time derivative that follows the parameterization ${\vec {x}}(\cdot , t)$ of ${\varGamma }(t)$, rather than ${\vec {u}}$. In particular, we let

3.3 $$\begin{aligned} \partial _t^\circ \, \zeta = \zeta _t + {\vec {\mathscr {V}}} \,.\,\nabla \,\zeta \qquad \forall \ \zeta \in H^1({\mathscr {G}_T}); \end{aligned}$$

where we stress once again that this definition is well-defined, even though $\zeta _t$ and $\nabla \,\zeta$ do not make sense separately for a function $\zeta \in H^1({\mathscr {G}_T})$. On recalling (2.7) we obtain that $\partial _t^\circ = \partial _t^\bullet$ if ${\vec {\mathscr {V}}} = {\vec {u}}$ on ${\varGamma }(t)$. Moreover, for later use we note that

3.4 $$\begin{aligned} \langle \zeta , \nabla _{\mathrm{s}}\,.\, {\vec {\eta }} \rangle _{{\varGamma }(t)} + \langle \nabla _{\mathrm{s}}\,\zeta , {\vec {\eta }} \rangle _{{\varGamma }(t)} = - \langle \zeta \,{\vec {\eta }} , {\vec {\varkappa }} \rangle _{{\varGamma }(t)} \qquad \forall \ \zeta \in H^1({\varGamma }(t)),\, {\vec {\eta }} \in [H^1({\varGamma }(t))]^d \end{aligned}$$

and

3.5 $$\begin{aligned} \frac{\mathrm{d}}{\mathrm{d}t}\left\langle \chi , \zeta \right\rangle _{{\varGamma }(t)} = \left\langle \partial _t^\circ \,\chi , \zeta \right\rangle _{{\varGamma }(t)} + \left\langle \chi , \partial _t^\circ \,\zeta \right\rangle _{{\varGamma }(t)} + \left\langle \chi \,\zeta , \nabla _{\mathrm{s}}\,.\,{\vec {\mathscr {V}}} \right\rangle _{{\varGamma }(t)} \quad \forall \ \chi ,\zeta \in H^1({\mathscr {G}_T}), \end{aligned}$$

see Definition 2.11 and Lemma 5.2 in [19], respectively.

The most natural weak formulation of the system (2.2a–d), (2.3), (2.4a–d), (2.5) uses the fluidic tangential velocity for the evolution of ${\varGamma }(t)$, and so (2.4d) is replaced by $\vec {\mathscr {V}} = {\vec {u}}$ on ${\varGamma }(t)$. It then follows from (2.4b), (2.9), and (3.4) that3.6where we have noted for symmetric matrices  that  for all . This weak formulation of the system (2.2a–d), (2.3), (2.4a–d), (2.5) is then given as follows. Find ${\varGamma }(t) = {\vec {x}}({\varUpsilon }, t)$ for $t\in [0,T]$ with ${\vec {\mathscr {V}}} \in [L^2({\mathscr {G}_T})]^d$ and ${\vec {\mathscr {V}}}(\cdot , t) \in [H^1({\varGamma }(t)]^d$ for almost all $t \in (0,T)$, and functions ${\vec {u}} \in \mathbb {V}_{\varGamma }({\vec {g}})$, $p \in L^2(0,T; {\widehat{\mathbb {P}}})$, $p_{\varGamma }\in L^2({\mathscr {G}_T})$, ${\vec {\varkappa }} \in [H^1({\mathscr {G}_T})]^d$ and ${\vec {f}}_{\varGamma }\in [L^2({\mathscr {G}_T})]^d$ such that for almost all $t \in (0,T)$ it holds that 3.7a
3.7b
3.7c
3.7d
3.7e
3.7f as well as the initial conditions (2.8), where in (3.7d) we have recalled (2.1). Here (3.7a–e) can be derived analogously to the weak formulation presented in [9, 11], recall (3.1), (3.2), (3.6) and (2.10). In addition, (3.7f) is based on (2.12).

In what follows we would like to derive an energy bound for a solution of (3.7a–f), where for ease of exposition we consider only the case ${\vec {g}} = {\vec {0}}$. All of the following considerations are formal, in the sense that we make the appropriate assumptions about the existence, boundedness and regularity of a solution to (3.7a–f). Firstly, it follows from (3.5), (3.7d) and (3.7c) with $\eta = |{\vec {u}} \mid _{{\varGamma }(t)}|^2$ that

3.8 $$\begin{aligned} \tfrac{1}{2}\,\rho _{\varGamma }\,\frac{\mathrm{d}}{\mathrm{d}t}\left\langle {\vec {u}}, {\vec {u}} \right\rangle _{{\varGamma }(t)}&= \tfrac{1}{2}\,\rho _{\varGamma }\left\langle \partial _t^\circ \,|{\vec {u}}|^2,1 \right\rangle _{{\varGamma }(t)} + \tfrac{1}{2}\,\rho _{\varGamma }\left\langle \nabla _{\mathrm{s}}\,.\,{\vec {\mathscr {V}}}, |{\vec {u}}|^2 \right\rangle _{{\varGamma }(t)} \nonumber \\&= \rho _{\varGamma }\left\langle \partial _t^\circ \,{\vec {u}}, {\vec {u}} \right\rangle _{{\varGamma }(t)} + \tfrac{1}{2}\,\rho _{\varGamma }\left\langle \nabla _{\mathrm{s}}\,.\, {\vec {u}}, |{\vec {u}}|^2 \right\rangle _{{\varGamma }(t)} = \rho _{\varGamma }\left\langle \partial _t^\circ \,{\vec {u}}, {\vec {u}} \right\rangle _{{\varGamma }(t)}. \end{aligned}$$

Now choosing ${\vec {\xi }} = {\vec {u}}$ in (3.7a), recall that ${\vec {g}} = {\vec {0}}$, $\varphi = p(\cdot , t)$ in (3.7b) and $\eta = p_{\varGamma }(\cdot , t)$ in (3.7c) yields, on combining with (3.8), that3.9Combining (3.9) with (2.12), on choosing ${\vec {\chi }} = {\vec {f}}_{\varGamma }$ in (3.7d) and ${\vec {\chi }} = {\vec {\mathscr {V}}}$ in (3.7f), yields that3.10Moreover, we note that it immediately follows from (3.5) and (3.7c, d) that

3.11 $$\begin{aligned} \frac{\mathrm{d}}{\mathrm{d}t} \, \mathscr {H}^{d-1} ({\varGamma }(t)) = \frac{\mathrm{d}}{\mathrm{d}t} \,\langle 1, 1 \rangle _{{\varGamma }(t)} = \left\langle 1, \nabla _{\mathrm{s}}\,.\,{\vec {\mathscr {V}}} \right\rangle _{{\varGamma }(t)} = \left\langle 1, \nabla _{\mathrm{s}}\,.\,{\vec {u}} \right\rangle _{{\varGamma }(t)} = 0. \end{aligned}$$

In addition, the volume of ${\varOmega }_-(t)$ is preserved in time, i.e. the mass of each phase is conserved. To see this, choose ${\vec {\chi }} = {\vec {\nu }}$ in (3.7d) and $\varphi = \left( \mathrm {\mathscr {X}}_{{\varOmega }_-(t)} - \frac{\mathscr {L}^d({\varOmega }_-(t))}{\mathscr {L}^d({\varOmega })}\right)$ in (3.7b) to obtain, on recalling [16, Lemma 2.1], that

3.12 $$\begin{aligned} \frac{\mathrm{d}}{\mathrm{d}t} \mathscr {L}^d({\varOmega }_-(t)) = \left\langle {\vec {\mathscr {V}}}, {\vec {\nu }} \right\rangle _{{\varGamma }(t)} = \left\langle {\vec {u}}, {\vec {\nu }} \right\rangle _{{\varGamma }(t)} = \int _{{\varOmega }_-(t)} \nabla \,.\,{\vec {u}} \;\mathrm{d}{\mathscr {L}}^{d} =0. \end{aligned}$$

Recalling the argument on the uniqueness of the pressures *p* and $p_{\varGamma }$ below (2.11a, b), we note the following LBB-type condition:3.13which we also refer to as the LBB$_{\varGamma }$ condition. Here we have defined the space , and let $\Vert {\vec {\eta }} \Vert _{1,{\varGamma }(t)}^2 := \left\langle {\vec {\eta }},{\vec {\eta }} \right\rangle _{{\varGamma }(t)} + \left\langle \nabla _{\mathrm{s}}\,{\vec {\eta }},\nabla _{\mathrm{s}}\,{\vec {\eta }} \right\rangle _{{\varGamma }(t)}$. In the case that the smooth hypersurface ${\varGamma }(t)$ is not a sphere, then (3.13) is shown to hold if $\partial _1{\varOmega }=\partial {\varOmega }$ is a smooth boundary in [28, p. 15].

## Semidiscrete finite element approximation

For simplicity we consider ${\varOmega }$ to be a polyhedral domain. Then let ${\mathscr {T}}^h$ be a regular partitioning of ${\varOmega }$ into disjoint open simplices $o^h_j$, $j = 1 ,\ldots , J_{\varOmega }$. Associated with ${\mathscr {T}}^h$ are the finite element spaces

$$\begin{aligned} S^h_k({\varOmega }) := \{\chi \in C(\overline{{\varOmega }}) : \chi \mid _{o} \in \mathscr {P}_k(o) \quad \forall \ o\in {\mathscr {T}}^h\} \subset H^1({\varOmega })\,, \qquad k \in {\mathbb N}, \end{aligned}$$

where $\mathscr {P}_k(o)$ denotes the space of polynomials of degree *k* on $o$. We also introduce $S^h_0({\varOmega })$, the space of piecewise constant functions on ${\mathscr {T}}^h$. Let $\{\varphi _{k,j}^h\}_{j=1}^{K_k^h}$ be the standard basis functions for $S^h_k({\varOmega })$, $k\ge 0$. We introduce ${\vec {I}}^h_k:[C(\overline{{\varOmega }})]^d\rightarrow [S^h_k({\varOmega })]^d$, $k\ge 1$, the standard interpolation operators, such that $({\vec {I}}^h_k\,{\vec {\eta }})({\vec {p}}_{k,j}^h)= {\vec {\eta }}({\vec {p}}_{k,j}^h)$ for $j=1,\ldots , K_k^h$; where $\{{\vec {p}}_{k,j}^h\}_{j=1}^{K_k^h}$ denotes the coordinates of the degrees of freedom of $S^h_k({\varOmega })$, $k\ge 1$. In addition we define the standard projection operator $I^h_0:L^1({\varOmega })\rightarrow S^h_0({\varOmega })$, such that

$$\begin{aligned} (I^h_0 \eta )\mid _{o} = \frac{1}{\mathscr {L}^d(o)}\,\int _{o} \eta \;\mathrm{d}{\mathscr {L}}^{d} \qquad \forall \ o \in \mathscr {T}^h. \end{aligned}$$

Our approximation to the velocity and pressure on ${\mathscr {T}}^h$ will be finite element spaces $\mathbb {U}^h({\vec {g}})\subset \mathbb {U}({\vec {I}}^h_k\,{\vec {g}})$, for some $k \ge 2$, where we assume from now on that ${\vec {g}} \in [C(\overline{{\varOmega }})]^d$, and $\mathbb {P}^h(t)\subset \mathbb {P}$. We require also the space ${\widehat{\mathbb {P}}}^h(t):= \mathbb {P}^h(t) \cap {\widehat{\mathbb {P}}}$. For the finite element spaces $(\mathbb {U}^h({\vec {g}}),\mathbb {P}^h)$ we may choose, for example, the lowest order Taylor–Hood element P2–P1, the P2–P0 element or the P2–(P1+P0) element on setting $\mathbb {U}^h({\vec {g}})=[S^h_2({\varOmega })]^d\cap \mathbb {U}({\vec {I}}^h_2\,{\vec {g}})$, and $\mathbb {P}^h = S^h_1({\varOmega }),\,S^h_0({\varOmega })$ or $S^h_1({\varOmega })+S^h_0({\varOmega })$, respectively. The lowest order Taylor–Hood element satisfies the standard LBB condition in the bulk for $d=2$ and $d=3$, while the other two choices satisfy it for $d=2$.

For the numerical approximation of the evolution of fluidic membranes it is desirable to maintain the surface area of the interface, recall (3.11), as well as the volume of the two phases, recall (3.12). In [8, 11] the present authors augmented the pressure space by the characteristic function of the inner phase in order to obtain discretizations that maintain the volume of the two phases. This enrichment of the pressure space is an example of an XFEM approach, and we refer to this particular approach as XFEM$_{\varGamma }$. Unfortunately, it does not appear possible to prove a discrete analogue of (3.11) for the XFEM$_{\varGamma }$ approach from [8, 11]. Hence in this paper we will modify the XFEM$_{\varGamma }$ approach so that we obtain numerical approximations that satisfy discrete analogues of both (3.12) and (3.11). From a practical point of view, this approach is very close to the procedure in [8, 11]. But the introduced modifications mean that the adjustments to the finite element approximations no longer have an interpretation within the XFEM framework. However, the new approach may be interpreted as an example of the recently proposed virtual element method, see below for further details.

The parametric finite element spaces in order to approximate e.g. ${\vec {\varkappa }}$ and $p_{\varGamma }$ are defined as follows, see also [5]. Let ${\varGamma }^h(t)\subset {\mathbb R}^d$ be a $(d-1)$-dimensional *polyhedral surface*, i.e. a union of non-degenerate $(d-1)$-simplices with no hanging vertices (see [16, p. 164] for $d=3$), approximating the closed surface ${\varGamma }(t)$. In particular, let ${\varGamma }^h(t)=\bigcup _{j=1}^{J_{\varGamma }} \overline{\sigma ^h_j(t)}$, where $\{\sigma ^h_j(t)\}_{j=1}^{J_{\varGamma }}$ is a family of mutually disjoint open $(d-1)$-simplices with vertices $\{{\vec {q}}^h_k(t)\}_{k=1}^{K_{\varGamma }}$. Then, for $k\in {\mathbb N}$, let

$$\begin{aligned} S^h_k({\varGamma }^h(t)) := \{\chi \in C({\varGamma }^h(t)) : \chi \mid _{\sigma ^h_j} \in \mathscr {P}_k(\sigma ^h_j) \quad \forall \ j=1,\ldots , J_{\varGamma }\} \subset H^1({\varGamma }^h(t)). \end{aligned}$$

We also introduce $S^h_0({\varGamma }^h(t))$, the space of piecewise constant functions on $\{\sigma ^h_j(t)\}_{j=1}^{J_{\varGamma }}$. For ease of presentation, we introduce the following notations for the spaces of piecewise linear functions on ${\varGamma }^h(t)$. Let $W({\varGamma }^h(t))= S^h_1({\varGamma }^h(t))$ and $\underline{V}({\varGamma }^h(t))= [S^h_1({\varGamma }^h(t))]^d$, with $\{\chi ^h_k(\cdot ,t)\}_{k=1}^{K_{\varGamma }}$ denoting the standard basis of $W({\varGamma }^h(t))$, i.e.

4.1 $$\begin{aligned} \chi ^h_k({\vec {q}}^h_l(t),t) = \delta _{kl}\quad \forall \ k,l \in \{1,\ldots ,K_{\varGamma }\}\,,\ t \in [0,T]. \end{aligned}$$

For later purposes, we also introduce the standard interpolation operators $\pi ^h_k(t):$ $C({\varGamma }^h(t))\rightarrow S^h_k({\varGamma }^h(t))$ and ${\vec {\pi }}^h_k(t): [C({\varGamma }^h(t))]^d\rightarrow [S^h_k({\varGamma }^h(t))]^d$, for $k\in {\mathbb N}$. For scalar and vector functions $\eta ,\zeta$ on ${\varGamma }^h(t)$ we introduce the $L^2$–inner product $\langle \cdot ,\cdot \rangle _{{\varGamma }^h(t)}$ over the polyhedral surface ${\varGamma }^h(t)$ as follows

$$\begin{aligned} \left\langle \eta , \zeta \right\rangle _{{\varGamma }^h(t)} := \int _{{\varGamma }^h(t)} \eta \,.\,\zeta \;\mathrm{d}{\mathscr {H}}^{d-1}. \end{aligned}$$

If *v*, *w* are piecewise continuous, with possible jumps across the edges of $\{\sigma _j^h\}_{j=1}^{J_{\varGamma }}$, we introduce the mass lumped inner product $\langle \cdot ,\cdot \rangle _{{\varGamma }^h(t)}^h$ as

$$\begin{aligned} \left\langle \eta , \zeta \right\rangle ^h_{{\varGamma }^h(t)} := \tfrac{1}{d} \sum _{j=1}^{J_{\varGamma }} \mathscr {H}^{d-1}(\sigma ^h_j)\,\sum _{k=1}^{d} (\eta \,.\,\zeta )(({\vec {q}}^h_{j_k})^-), \end{aligned}$$

where $\{{\vec {q}}^h_{j_k}\}_{k=1}^{d}$ are the vertices of $\sigma ^h_j$, and where we define $\eta (({\vec {q}}^h_{j_k})^-):= {\mathop {\hbox {lim}}\limits _{\sigma ^h_j\ni {\vec {p}}\rightarrow {\vec {q}}^h_{j_k}}\, \eta ({\vec {p}})}$.

Following [19, (5.23)], we define the discrete material velocity for ${\vec {z}} \in {\varGamma }^h(t)$ by

4.2 $$\begin{aligned} {\vec {\mathscr {V}}}^h({\vec {z}}, t) := \sum _{k=1}^{K_{\varGamma }} \left[ \frac{\mathrm{d}}{\mathrm{d}t}\,{\vec {q}}^h_k(t)\right] \chi ^h_k({\vec {z}}, t). \end{aligned}$$

Then we define, similarly to (3.3),

4.3 $$\begin{aligned} \partial _t^{\circ ,h}\, \zeta = \zeta _t + {\vec {\mathscr {V}}}^h\,.\,\nabla \,\zeta \qquad \forall \ \zeta \in H^1({\mathscr {G}^h_T})\,, \quad \text {where}\quad {\mathscr {G}^h_T}:= \bigcup _{t \in [0,T]} {\varGamma }^h(t) \times \{t\}. \end{aligned}$$

For later use, we also introduce the finite element spaces

$$\begin{aligned} W({\mathscr {G}^h_T})&:= \{ \chi \in C({\mathscr {G}^h_T}) : \chi (\cdot , t) \in W({\varGamma }^h(t))\quad \forall \ t \in [0,T] \}, \\ W_T({\mathscr {G}^h_T})&:= \{ \chi \in W({\mathscr {G}^h_T}) : \partial _t^{\circ ,h}\,\chi \in C({\mathscr {G}^h_T}) \}, \end{aligned}$$

as well as

$$\begin{aligned} \mathbb {V}^h_{{\varGamma }^h}({\vec {g}}) := \{ {\vec {\phi }} \in H^1(0,T; \mathbb {U}^h({\vec {g}}))&: \exists \ {\vec {\chi }} \in [W_T({\mathscr {G}^h_T})]^d, \\&\quad \text { s.t. } {\vec {\chi }}(\cdot ,t) = {\vec {\pi }}^h_1\,[{\vec {\phi }}\mid _{{\varGamma }^h(t)}]\ \quad \forall \ t\in [0,T]\}. \end{aligned}$$

On differentiating (4.1) with respect to *t*, it immediately follows that

4.4 $$\begin{aligned} \partial _t^{\circ ,h}\, \chi ^h_k = 0 \quad \forall \ k \in \{1,\ldots ,K_{\varGamma }\}, \end{aligned}$$

see [19, Lem. 5.5]. It follows directly from (4.4) that

$$\begin{aligned} \partial _t^{\circ ,h}\,\zeta (\cdot ,t) = \sum _{k=1}^{K_{\varGamma }} \chi ^h_k(\cdot ,t)\, \frac{\mathrm{d}}{\mathrm{d}t}\,\zeta _k(t) \quad \text {on}\ {\varGamma }^h(t) \end{aligned}$$

for $\zeta (\cdot ,t) = \sum _{k=1}^{K_{\varGamma }} \zeta _k(t)\,\chi ^h_k(\cdot ,t) \in W({\varGamma }^h(t))$, and hence $\partial _t^{\circ ,h}\,{\vec {\mathrm{id}}} = {\vec {\mathscr {V}}}^h$ on ${\varGamma }^h(t)$.

We recall from [19, Lem. 5.6] that

4.5 $$\begin{aligned} \frac{\mathrm{d}}{\mathrm{d}t}\, \int _{\sigma ^h_j(t)} \zeta \;\mathrm{d}{\mathscr {H}}^{d-1} = \int _{\sigma ^h_j(t)} \partial _t^{\circ ,h}\,\zeta + \zeta \,\nabla _{\mathrm{s}}\,.\,{\vec {\mathscr {V}}}^h \;\mathrm{d}{\mathscr {H}}^{d-1} \quad \forall \ \zeta \in H^1(\sigma ^h(t)), \end{aligned}$$

for $j = 1,\ldots ,J_{\varGamma }$, which immediately implies that

4.6 $$\begin{aligned}&\frac{\mathrm{d}}{\mathrm{d}t}\langle \eta , \zeta \rangle _{{\varGamma }^h(t)} = \langle \partial _t^{\circ ,h}\,\eta , \zeta \rangle _{{\varGamma }^h(t)} + \langle \eta , \partial _t^{\circ ,h}\,\zeta \rangle _{{\varGamma }^h(t)} + \langle \eta \,\zeta , \nabla _{\mathrm{s}}\,.\,{\vec {\mathscr {V}}}^h \rangle _{{\varGamma }^h(t)} \nonumber \\& \qquad \forall \ \eta ,\zeta \in W_T({\mathscr {G}^h_T}). \end{aligned}$$

Similarly, we recall from [9, Lem. 3.1] that

4.7 $$\begin{aligned}&\frac{\mathrm{d}}{\mathrm{d}t}\langle \eta , \zeta \rangle _{{\varGamma }^h(t)}^h = \langle \partial _t^{\circ ,h}\,\eta , \zeta \rangle _{{\varGamma }^h(t)}^h + \langle \eta , \partial _t^{\circ ,h}\,\zeta \rangle _{{\varGamma }^h(t)}^h + \langle \eta \,\zeta , \nabla _{\mathrm{s}}\,.\,{\vec {\mathscr {V}}}^h \rangle _{{\varGamma }^h(t)}^h \nonumber \\& \qquad \forall \ \eta ,\zeta \in W_T({\mathscr {G}^h_T}). \end{aligned}$$

Given ${\varGamma }^h(t)$, we let ${\varOmega }^h_+(t)$ denote the exterior of ${\varGamma }^h(t)$ and let ${\varOmega }^h_-(t)$ denote the interior of ${\varGamma }^h(t)$, so that ${\varGamma }^h(t) = \partial {\varOmega }^h_-(t) = \overline{{\varOmega }^h_-(t)} \cap \overline{{\varOmega }^h_+(t)}$. We then partition the elements of the bulk mesh $\mathscr {T}^h$ into interior, exterior and interfacial elements as follows. Let

$$\begin{aligned} \mathscr {T}^h_-(t)&:= \{ o \in \mathscr {T}^h : o \subset {\varOmega }^h_-(t) \}, \nonumber \\ \mathscr {T}^h_+(t)&:= \{ o \in \mathscr {T}^h : o \subset {\varOmega }^h_+(t) \}, \nonumber \\ \mathscr {T}^h_{{\varGamma }^h}(t)&:= \{ o \in \mathscr {T}^h : o \cap {\varGamma }^h(t) \not = \emptyset \}. \end{aligned}$$

Clearly $\mathscr {T}^h = \mathscr {T}^h_-(t) \cup \mathscr {T}^h_+(t) \cup \mathscr {T}^h_{{\varGamma }}(t)$ is a disjoint partition. In addition, we define the piecewise constant unit normal ${\vec {\nu }}^h(t)$ to ${\varGamma }^h(t)$ such that ${\vec {\nu }}^h(t)$ points into ${\varOmega }^h_+(t)$. Moreover, we introduce the discrete density $\rho ^h(t) \in S^h_0({\varOmega })$ and the discrete viscosity $\mu ^h(t) \in S^h_0({\varOmega })$ as

$$\begin{aligned} \rho ^h(t)\mid _{o} = {\left\{ \begin{array}{ll} \rho _- &{} o \in \mathscr {T}^h_-(t), \\ \rho _+ &{} o \in \mathscr {T}^h_+(t), \\ \tfrac{1}{2}\,(\rho _- + \rho _+) &{} o \in \mathscr {T}^h_{{\varGamma }^h}(t), \end{array}\right. } \ \text {and }\ \mu ^h(t)\mid _{o} = {\left\{ \begin{array}{ll} \mu _- &{} o \in \mathscr {T}^h_-(t), \\ \mu _+ &{} o \in \mathscr {T}^h_+(t), \\ \tfrac{1}{2}\,(\mu _- + \mu _+) &{} o \in \mathscr {T}^h_{{\varGamma }^h}(t). \end{array}\right. } \end{aligned}$$

We introduce, similarly to (2.6a, b), 4.8aand4.8b where here  denotes the surface gradient on ${\varGamma }^h(t)$.

In what follows we will introduce a finite element approximation for the free boundary problem (2.2a–d), (2.3), (2.4a–d), (2.5). Here ${\vec {U}}^h(\cdot , t) \in \mathbb {U}^h({\vec {g}})$ will be an approximation to ${\vec {u}}(\cdot , t)$, while $P^h(\cdot , t) \in {\widehat{\mathbb {P}}}^h(t)$ approximates $p(\cdot , t)$ and $P_{\varGamma }^h(\cdot , t) \in W({\varGamma }^h(t))$ approximates $p_{\varGamma }(\cdot , t)$. When designing such a finite element approximation, a careful decision has to be made about the *discrete tangential velocity* of ${\varGamma }^h(t)$. The most natural choice is to select the velocity of the fluid, i.e. (3.7d) is appropriately discretized, and that is the approach we adopt in this paper. Overall, we then obtain the following semidiscrete continuous-in-time finite element approximation, which is the semidiscrete analogue of the weak formulation (3.7a–f).

Given $\ell \in \{1,2\}$, ${\varGamma }^h(0)$ and ${\vec {U}}^h(\cdot ,0) \in \mathbb {U}^h({\vec {g}})$, find ${\varGamma }^h(t)$ such that ${\vec {\mathrm{id}}} \mid _{{\varGamma }^h(t)} \in \underline{V}({\varGamma }^h(t))$ for $t \in [0,T]$, and functions ${\vec {U}}^h \in \mathbb {V}^h_{{\varGamma }^h}({\vec {g}})$, $P^h \in \mathbb {P}^h_T := \{ \varphi \in L^2(0,T; {\widehat{\mathbb {P}}}) : \varphi (t) \in {\widehat{\mathbb {P}}}^h(t) \text { for a.e. } t \in (0,T)\}$, $P_{\varGamma }^h \in W({\mathscr {G}^h_T})$, ${\vec {\kappa }}^h \in [W({\mathscr {G}^h_T})]^d$ and ${\vec {F}}_{\varGamma }^h \in [W({\mathscr {G}^h_T})]^d$ such that for almost all $t \in (0,T)$ it holds that 4.9a
4.9b
4.9c
4.9d
4.9e
4.9f where we recall (4.2). Here we have defined ${\vec {f}}^h(\cdot ,t) := {\vec {I}}^h_2\,{\vec {f}}(\cdot ,t)$, where here and throughout we assume that ${\vec {f}} \in L^2(0,T;[C(\overline{{\varOmega }})]^d)$. We observe that (4.9d) collapses to ${\vec {\mathscr {V}}}^h = {\vec {\pi }}^h_1\, {\vec {U}}^h\mid _{{\varGamma }^h(t)} \in \underline{V}({\varGamma }^h(t))$, which on recalling (4.3) turns out to be crucial for the stability analysis for (4.9a–f). It is for this reason that we use mass lumping in (4.9d). The superscript $\cdot ^{(h)}$ in (4.9e, f) means that we can consider the corresponding terms either with or without mass lumping. Here we note that the scheme (4.9d–f), with true integration used throughout, and with ${\vec {U}}^h$ in (4.9d) replaced by ${\vec {F}}^h_{\varGamma }$, is the stable approximation of Willmore flow from [18], see also [15] for the case $d=2$. In fact, for $d=2$ we observe thatwhere $\cdot _\mathrm{s}$ in the last term denotes differentiation with respect to arclength; compare also [7, (3.12 a, b)].

In the following theorem we derive discrete analogues of (3.10) and (3.11) for the scheme (4.9a–f).

### **Theorem 4.1**

Let $\{({\varGamma }^h, {\vec {U}}^h, P^h, P^h_{\varGamma }, {\vec {\kappa }}^h, {\vec {F}}_{\varGamma }^h)(t) \}_{t\in [0,T]}$ be a solution to (4.9a–f). Then, in the case ${\vec {g}} = {\vec {0}}$, and if $(\ell - 1)\,\rho _{\varGamma }= 0$, it holds that4.10If $\ell = 1$, it also holds that

4.11 $$\begin{aligned} \frac{\mathrm{d}}{\mathrm{d}t}\left\langle \chi ^h_k, 1 \right\rangle _{{\varGamma }^h(t)} = 0 \quad \forall \ k \in \{1,\ldots ,K_{\varGamma }\} \end{aligned}$$

and hence that

4.12 $$\begin{aligned} \frac{\mathrm{d}}{\mathrm{d}t}\, \mathscr {H}^{d-1} ({\varGamma }^h(t)) = 0. \end{aligned}$$

### *Proof*

Choosing ${\vec {\xi }} = {\vec {U}}^h$ in (4.9a), recall that ${\vec {g}} = {\vec {0}}$, $\varphi = P^h$ in (4.9b) and $\eta = P^h_{\varGamma }$ in (4.9c) yields that4.13Moreover, it is possible to show, similarly to (2.12), thatsee [18]. Hence choosing ${\vec {\chi }} = {\vec {F}}^h_{\varGamma }$ in (4.9d) and ${\vec {\chi }} = {\vec {\mathscr {V}}}^h$ in (4.9f) yields that

4.14 $$\begin{aligned} \left\langle {\vec {F}}_{\varGamma }^h,{\vec {U}}^h \right\rangle _{{\varGamma }^h(t)}^h = \left\langle {\vec {F}}_{\varGamma }^h,{\vec {\mathscr {V}}}^h \right\rangle _{{\varGamma }^h(t)}^h = -\tfrac{1}{2}\,\frac{\mathrm{d}}{\mathrm{d}t}\left\langle {\vec {\kappa }}^h, {\vec {\kappa }}^h\right\rangle _{{\varGamma }^h(t)}^{(h)}. \end{aligned}$$

If $\rho _{\varGamma }= 0$, then the desired result (4.10) directly follows from combining (4.13) and (4.14). If $\rho _{\varGamma }> 0$, on the other hand, then the assumptions mean that $\ell =1$. Then we note, similarly to (3.8), that (4.7), (4.9d) and (4.9c) with $\eta = \pi ^h_1\,[|{\vec {U}}^h \mid _{{\varGamma }^h(t)}|^2]$ imply that

4.15 $$\begin{aligned} \tfrac{1}{2}\,\rho _{\varGamma }\,\frac{\mathrm{d}}{\mathrm{d}t}\left\langle {\vec {U}}^h, {\vec {U}}^h \right\rangle _{{\varGamma }^h(t)}^h&= \tfrac{1}{2}\,\rho _{\varGamma }\left\langle \partial _t^{\circ ,h}\,{\vec {\pi }}^h_1\,[|{\vec {U}}^h|^2],1 \right\rangle _{{\varGamma }^h(t)}^h + \tfrac{1}{2}\,\rho _{\varGamma }\left\langle \nabla _{\mathrm{s}}\,.\,{\vec {\mathscr {V}}}^h, |{\vec {U}}^h|^2 \right\rangle _{{\varGamma }^h(t)}^h \nonumber \\&= \rho _{\varGamma }\left\langle \partial _t^{\circ ,h}\,{\vec {\pi }}^h_1\,{\vec {U}}^h, {\vec {U}}^h \right\rangle _{{\varGamma }^h(t)}^h + \tfrac{1}{2}\,\rho _{\varGamma }\left\langle \nabla _{\mathrm{s}}\,.\,({\vec {\pi }}^h_1\, {\vec {U}}^h), |{\vec {U}}^h|^2 \right\rangle _{{\varGamma }^h(t)}^h \nonumber \\&= \rho _{\varGamma }\left\langle \partial _t^{\circ ,h}\,{\vec {\pi }}^h_1\,{\vec {U}}^h, {\vec {U}}^h \right\rangle _{{\varGamma }^h(t)}^h. \end{aligned}$$

Combining (4.13), (4.15) and (4.14) yields the desired result (4.10) for $\rho _{\varGamma }> 0$.

In the case $\ell = 1$, it immediately follows from (4.6) and (4.4), similarly to (3.11), on choosing $\eta = \chi ^h_k$ in (4.9c), and on recalling from (4.9d) that ${\vec {\mathscr {V}}}^h = {\vec {\pi }}^h_1\,{\vec {U}}^h\mid _{{\varGamma }^h(t)}$, that

4.16 $$\begin{aligned} \frac{\mathrm{d}}{\mathrm{d}t}\left\langle \chi ^h_k, 1 \right\rangle _{{\varGamma }^h(t)} = \left\langle \chi ^h_k, \nabla _{\mathrm{s}}\,.\,{\vec {\mathscr {V}}}^h \right\rangle _{{\varGamma }^h(t)} = \left\langle \chi ^h_k, \nabla _{\mathrm{s}}\,.\, ({\vec {\pi }}^h_1\,{\vec {U}}^h ) \right\rangle _{{\varGamma }^h(t)} = 0, \end{aligned}$$

which proves the desired result (4.11). Summing (4.11) for all $k = 1,\ldots ,K_{\varGamma }$ then yields the desired result (4.12). $\square$

### *Remark 4.1*

We remark that (4.11) ensures that the measure of the support of each basis function on ${\varGamma }^h(t)$ is conserved. In the case $d=2$, and for $J_{\varGamma }$ being odd, this is equivalent to each element $\sigma ^h_j$ maintaining its length. In particular, if ${\varGamma }^h(0)$ is equidistributed, then ${\varGamma }^h(t)$ will remain equidistributed throughout.

The same result, for arbitrary $J_{\varGamma }\ge 2$ and for $d=2$ and $d=3$, can be obtained on replacing the space of continuous piecewise linear finite elements $W({\varGamma }^h(t))$ for the surface pressure functions $P^h_{\varGamma }$, and for the test functions in (4.9c), with the space of discontinuous piecewise constant functions, $S^h_0({\varGamma }^h(t))$. Then (4.5), similarly to (4.16), immediately yields that

4.17 $$\begin{aligned} \frac{\mathrm{d}}{\mathrm{d}t}\,\mathscr {H}^{d-1}(\sigma ^h_j(t)) = 0 \qquad \forall \ j \in \{1,\ldots ,J_{\varGamma }\}. \end{aligned}$$

While this property may appear desirable at first, our numerical experience for the fully discrete variant of this modified (4.9a–f) indicates that in the case $d=3$ the constraint (4.17) is too severe. For example, the evolution for the fully discrete analogues of ${\varGamma }^h(t)$ may lag behind the observed evolution for the equivalent simulation with $S^h_0({\varGamma }^h(t))$ replaced by $W({\varGamma }^h(t))$. This can even lead to locking, where the linear solvers are no longer able to find a discrete solution at a certain time step. Here we note that for typical triangulations of ${\varGamma }^h(t)$ it holds that $J_{\varGamma }\approx 2\,K_{\varGamma }$. It is for this reason that we prefer the scheme (4.9a–f) as stated.

### *Remark 4.2*

For the proofs of (4.11) and (4.12) it is crucial that (4.9c) holds with linear interpolation, i.e. $\ell = 1$. Similarly, for the proof of (4.10) with $\rho _{\varGamma }>0$ it is necessary to choose $\ell = 1$. For the surface Navier–Stokes system, this means that we use linear velocity approximations with linear pressures, something that is unlikely to satisfy a discrete surface LBB condition. In fact, in practice this can lead to an oscillatory approximation of the surface pressure, as can be seen in Fig. 5 below.

This can be avoided by using a quadratic interpolation of the bulk velocities, i.e. choosing $\ell = 2$. This gives better behaved surface pressure approximations in practice, but the mesh quality is no longer maintained. Moreover, it can no longer be shown that (4.12) holds. However, in practice the evolutions of the fully discrete analogues of ${\varGamma }^h(t)$ are nearly identical for the two cases $\ell = 1$ and $\ell = 2$, at least when $d=2$. When $\ell = 2$ that means that in practice the surface area is maintained well, while for $\ell =1$ it means that despite the oscillatory surface pressures, the approximation of the velocity is well-behaved. Note that in three dimensional simulations we observe that the scheme with $\ell = 2$ does not conserve the overall surface area well. Hence it does not appear to be a viable scheme in practice.

We observe that it does not appear possible to prove a discrete analogue of (3.12) for the scheme (4.9a–f). The reason is that ${\vec {\chi }} = {\vec {\nu }}^h$ is not a valid test function in (4.9d). However, a procedure similar to the XFEM$_{\varGamma }$ approach introduced by the authors in [8, 11] ensures that a modified variant of (4.9a–f) conserves the enclosed volumes. To this end, we introduce the vertex normal function ${\vec {\omega }}^h(\cdot , t) \in \underline{V}({\varGamma }^h(t))$ with

$$\begin{aligned} {\vec {\omega }}^h({\vec {q}}^h_k(t),t ) := \frac{1}{\mathscr {H}^{d-1}({\varLambda }^h_k(t))} \sum _{j\in {\varTheta }_k^h} \mathscr {H}^{d-1}(\sigma ^h_j(t))\, {\vec {\nu }}^h\mid _{\sigma ^h_j(t)}, \end{aligned}$$

where for $k= 1 ,\ldots , K_{\varGamma }^h$ we define ${\varTheta }_k^h:= \{j : {\vec {q}}^h_k(t) \in \overline{\sigma ^h_j(t)}\}$ and set ${\varLambda }_k^h(t) :=$ $\cup _{j \in {\varTheta }_k^h} \overline{\sigma ^h_j(t)}$. For later use we note that

4.18 $$\begin{aligned} \left\langle {\vec {z}}, w\,{\vec {\nu }}^h\right\rangle _{{\varGamma }^h(t)}^h = \left\langle {\vec {z}}, w\,{\vec {\omega }}^h\right\rangle _{{\varGamma }^h(t)}^h \qquad \forall \ {\vec {z}} \in \underline{V}({\varGamma }^h(t))\,,\ w \in W({\varGamma }^h(t)). \end{aligned}$$

We are now in a position to propose the following adaptation of (4.9a–f).

Given $\ell \in \{1,2\}$, ${\varGamma }^h(0)$ and ${\vec {U}}^h(\cdot ,0) \in \mathbb {U}^h({\vec {g}})$, find ${\varGamma }^h(t)$ such that ${\vec {\mathrm{id}}} \mid _{{\varGamma }^h(t)} \in \underline{V}({\varGamma }^h(t))$ for $t \in [0,T]$, and functions ${\vec {U}}^h \in \mathbb {V}^h_{{\varGamma }^h}({\vec {g}})$, $P^h \in \mathbb {P}^h_T$, $P_\mathrm{sing}^h \in L^2(0,T; {\mathbb R})$, $P_{\varGamma }^h \in W({\mathscr {G}^h_T})$, ${\vec {\kappa }}^h \in [W({\mathscr {G}^h_T})]^d$ and ${\vec {F}}_{\varGamma }^h \in [W({\mathscr {G}^h_T})]^d$ such that for almost all $t \in (0,T)$ it holds that 4.19a
4.19b and (4.9c–f) hold. Of course, ${\vec {\chi }} = {\vec {\omega }}^h$ is a valid test function in (4.9d), and so combining with (4.19b) yields a discrete volume preservation property, as is shown in the following theorem.

### **Theorem 4.2**

Let $\{({\varGamma }^h,{\vec {U}}^h, P^h, P_\mathrm{sing}^h, P^h_{\varGamma }, {\vec {\kappa }}^h, {\vec {F}}_{\varGamma }^h)(t) \}_{t\in [0,T]}$ be a solution to (4.19a, b), (4.9c–f). Then (4.10) holds if ${\vec {g}} = {\vec {0}}$ and $(\ell -1)\,\rho _{\varGamma }=0$. In addition, (4.12) holds for $\ell = 1$, while

4.20 $$\begin{aligned} \frac{\mathrm{d}}{\mathrm{d}t}\, \mathscr {L}^d({\varOmega }_-^h(t)) = 0 \end{aligned}$$

holds for $$\ell \in \{1,2\}$$.

### *Proof*

The proofs for (4.10) and (4.12) are analogous to the proofs in Theorem 4.1. In order to prove (4.20) we choose ${\vec {\chi }} = {\vec {\omega }}^h \in \underline{V}({\varGamma }^h(t))$ in (4.9d) to yield that

$$\begin{aligned} \frac{\mathrm{d}}{\mathrm{d}t} \mathscr {L}^d({\varOmega }_-^h(t))&= \left\langle {\vec {\mathscr {V}}}^h , {\vec {\nu }}^h \right\rangle _{{\varGamma }^h(t)} = \left\langle {\vec {\mathscr {V}}}^h , {\vec {\nu }}^h \right\rangle ^h_{{\varGamma }^h(t)} = \left\langle {\vec {\mathscr {V}}}^h , {\vec {\omega }}^h \right\rangle ^h_{{\varGamma }^h(t)} = \left\langle {\vec {U}}^h, {\vec {\omega }}^h \right\rangle _{{\varGamma }^h(t)}^h \\&=0, \end{aligned}$$

where we have used (4.18) and (4.19b). $\square$

In order to interpret the adaptation in (4.19a, b) physically, we note the following. Of course, $P^h$ and $P_\mathrm{sing}^h$ act as Lagrange multipliers for the conditions in (4.19b). Moreover, on noting that

$$\begin{aligned} \left( \nabla \,.\,{\vec {\xi }}, \mathrm {\mathscr {X}}_{{\varOmega }_-^h(t)} \right) = \int _{{\varOmega }_-^h(t)} \nabla \,.\,{\vec {\xi }} \;\mathrm{d}{\mathscr {L}}^{d} = \left\langle {\vec {\xi }}, {\vec {\nu }}^h \right\rangle _{{\varGamma }^h(t)} \qquad \forall \ {\vec {\xi }} \in \mathbb {U}^h({\vec {0}}), \end{aligned}$$

we observe that replacing

4.21 $$\begin{aligned} \left\langle {\vec {\omega }}^h, {\vec {\xi }} \right\rangle _{{\varGamma }^h(t)}^h \quad \text {and}\quad \left\langle {\vec {U}}^h , {\vec {\omega }}^h \right\rangle _{{\varGamma }^h(t)}^h \end{aligned}$$

in (4.19a, b) by

$$\begin{aligned} \left\langle {\vec {\nu }}^h, {\vec {\xi }} \right\rangle _{{\varGamma }^h(t)} \quad \text {and}\quad \left\langle {\vec {U}}^h , {\vec {\nu }}^h \right\rangle _{{\varGamma }^h(t)} \end{aligned}$$

corresponds to augmenting $\mathbb {P}^h$ in (4.9a, b) with the single additional basis function $\mathrm {\mathscr {X}}_{{\varOmega }_-^h(t)}$. This is the XFEM$_{\varGamma }$ approach introduced by the authors in [8, 11], which for the schemes introduced there naturally leads to the conservation of the volume of the two phases. Such an XFEM interpretation is no longer possible for the modifications (4.21), as one cannot identify the corresponding additional basis function in the bulk. Therefore this can be viewed as an example of the recently proposed framework of virtual element methods, see e.g. [41]. In addition, on recalling (4.18) we have for a fixed time $t\in [0,T]$ that

4.22 $$\begin{aligned}&\left| \left\langle {\vec {\omega }}^h, {\vec {\xi }} \right\rangle _{{\varGamma }^h(t)}^h - \left\langle {\vec {\nu }}^h, {\vec {\xi }} \right\rangle _{{\varGamma }^h(t)} \right| = \left| \left\langle {\vec {\nu }}^h, {\vec {\pi }}^h_1\,{\vec {\xi }} - {\vec {\xi }} \right\rangle _{{\varGamma }^h(t)} \right| \nonumber \\&\qquad \qquad \le \left\langle |{\vec {\pi }}^h_1\,{\vec {\xi }} - {\vec {\xi }}|, 1 \right\rangle _{{\varGamma }^h(t)} \rightarrow 0 \quad \text {as}\quad h_{\varGamma }(t) \rightarrow 0 \qquad \forall \ {\vec {\xi }} \in [C(\overline{{\varOmega }})]^d, \end{aligned}$$

if $\mathscr {H}^{d-1} ( {\varGamma }^h(t))$ remains bounded as $h_{\varGamma }(t) \rightarrow 0$, where $h_{\varGamma }(t) := \max _{j=1 ,\ldots , J_{\varGamma }}$ $\text{ diam }( \sigma ^h_j(t) )$. It follows from (4.22) that we can interpret $P^h(\cdot , t) + P_\mathrm{sing}^h(t)\,\mathrm {\mathscr {X}}_{{\varOmega }_-^h(t)}$ as the natural approximation to the pressure $p(\cdot ,t)$ arising from (4.19a, b), (4.9c–f).

## Fully discrete finite element approximation

In this section we consider a fully discrete variant of the scheme (4.19a, b), (4.9c–f) from §4. Here we will choose the time discretization such that existence and uniqueness of the discrete solutions can be guaranteed, and such that we inherit as much of the structure of the stable schemes in [8, 11] as possible, see below for details.

We consider the partitioning $t_m = m\,\tau$, $m = 0,\ldots , M$, of [0, *T*] into uniform time steps $\tau = T / M$. The time discrete spatial discretizations then directly follow from the finite element spaces introduced in Sect. 3, where in order to allow for adaptivity in space we consider bulk finite element spaces that change in time. For all $m\ge 0$, let $\mathscr {T}^m$ be a regular partitioning of ${\varOmega }$ into disjoint open simplices $o^m_j$, $j = 1 ,\ldots , J^m_{\varOmega }$. Associated with ${\mathscr {T}}^m$ are the finite element spaces $S^m_k({\varOmega })$ for $k\ge 0$. We introduce also ${\vec {I}}^m_k:[C(\overline{{\varOmega }})]^d\rightarrow [S^m_k({\varOmega })]^d$, $k\ge 1$, the standard interpolation operators, and the standard projection operator $I^m_0:L^1({\varOmega })\rightarrow S^m_0({\varOmega })$. Similarly, the parametric finite element spaces are given by

$$\begin{aligned} S^m_k({\varGamma }^m) := \{\chi \in C({\varGamma }^m):{\vec {\chi }}\mid _{\sigma ^m_j} \in \mathscr {P}_k(\sigma ^m_j) \quad \forall \ j=1,\ldots , J_{\varGamma }\} \end{aligned}$$

for $m=0 ,\ldots , M-1$ and $k \in {\mathbb N}$. Here ${\varGamma }^m=\bigcup _{j=1}^{J_{\varGamma }} \overline{\sigma ^m_j}$, where $\{\sigma ^m_j\}_{j=1}^{J_{\varGamma }}$ is a family of mutually disjoint open $(d-1)$-simplices with vertices $\{{\vec {q}}^m_k\}_{k=1}^{K_{\varGamma }}$. For ease of notation we set $W({\varGamma }^m)= S^m_1({\varGamma }^m)$ and $\underline{V}({\varGamma }^m)= [S^m_1({\varGamma }^m)]^d$. We denote the standard basis of $W({\varGamma }^m)$ by $\{\chi ^m_k(\cdot ,t)\}_{k=1}^{K_{\varGamma }}$. We also introduce the standard interpolation operators $\pi ^m_k: C({\varGamma }^m)\rightarrow S^m_k({\varGamma }^m)$ and ${\vec {\pi }}^m_k: [C({\varGamma }^m)]^d\rightarrow [S^m_k({\varGamma }^m)]^d$, for $k \in {\mathbb N}$. Throughout this paper, we will parameterize the new closed surface ${\varGamma }^{m+1}$ over ${\varGamma }^m$, with the help of a parameterization ${\vec {X}}^{m+1} \in \underline{V}({\varGamma }^m)$, i.e. ${\varGamma }^{m+1} = {\vec {X}}^{m+1}({\varGamma }^m)$.

Given ${\varGamma }^m$, we let ${\varOmega }^m_+$ denote the exterior of ${\varGamma }^m$ and let ${\varOmega }^m_-$ denote the interior of ${\varGamma }^m$, so that ${\varGamma }^m = \partial {\varOmega }^m_- = \overline{{\varOmega }^m_-} \cap \overline{{\varOmega }^m_+}$. In addition, we define the piecewise constant unit normal ${\vec {\nu }}^m$ to ${\varGamma }^m$ such that ${\vec {\nu }}^m$ points into ${\varOmega }^m_+$. We then partition the elements of the bulk mesh $\mathscr {T}^m$ into interior, exterior and interfacial elements as before, and we introduce $\rho ^m,\,\mu ^m \in S^m_0({\varOmega })$, for $m\ge 0$, as

$$\begin{aligned} \rho ^m\mid _{o^m} = {\left\{ \begin{array}{ll} \rho _- &{} o^m \in \mathscr {T}^m_-, \\ \rho _+ &{} o^m \in \mathscr {T}^m_+, \\ \tfrac{1}{2}\,(\rho _- + \rho _+) &{} o^m \in \mathscr {T}^m_{{\varGamma }^m}, \end{array}\right. } \quad \text {and}\quad \mu ^m\mid _{o^m} = {\left\{ \begin{array}{ll} \mu _- &{} o^m \in \mathscr {T}^m_-, \\ \mu _+ &{} o^m \in \mathscr {T}^m_+, \\ \tfrac{1}{2}\,(\mu _- + \mu _+) &{} o^m \in \mathscr {T}^m_{{\varGamma }^m}. \end{array}\right. } \end{aligned}$$

We also introduce the $L^2$–inner product $\langle \cdot ,\cdot \rangle _{{\varGamma }^m}$ over the current polyhedral surface ${\varGamma }^m$, as well as the the mass lumped inner product $\langle \cdot ,\cdot \rangle _{{\varGamma }^m}^h$. We introduce, similarly to (4.8a, b),andwhere here  denotes the surface gradient on ${\varGamma }^m$.

We introduce the following pushforward operator for the discrete interfaces ${\varGamma }^m$ and ${\varGamma }^{m-1}$, for $m=0,\ldots ,M$. Here we set ${\varGamma }^{-1}:={\varGamma }^0$. Let ${\vec {{\varPi }}}_{m-1}^m : [C({\varGamma }^{m-1})]^d \rightarrow \underline{V}({\varGamma }^m)$ such that

5.1 $$\begin{aligned} ({\vec {{\varPi }}}_{m-1}^m\,{\vec {z}})({\vec {q}}^m_k) = {\vec {z}}({\vec {q}}^{m-1}_k), \qquad k = 1,\ldots ,K_{\varGamma }\,,\qquad \forall \ {\vec {z}} \in [C({\varGamma }^{m-1})]^d, \end{aligned}$$

for $m=1,\ldots ,M$, and set ${\vec {{\varPi }}}_{-1}^0 := {\vec {\pi }}^0_1$. Analogously to (5.1) we also introduce ${\varPi }_{m-1}^m : C({\varGamma }^{m-1}) \rightarrow W({\varGamma }^m)$. We note, similarly to (4.18), that

$$\begin{aligned} \left\langle {\vec {z}}, w\,{\vec {\nu }}^m\right\rangle _{{\varGamma }^m}^h = \left\langle {\vec {z}}, w\,{\vec {\omega }}^m\right\rangle _{{\varGamma }^m}^h \qquad \forall \ {\vec {z}} \in \underline{V}({\varGamma }^m)\,,\ w \in W({\varGamma }^m), \end{aligned}$$

where ${\vec {\omega }}^m := \sum _{k=1}^{K_{\varGamma }} \chi ^m_k\,{\vec {\omega }}^m_k \in \underline{V}({\varGamma }^m)$, and where for $k= 1 ,\ldots , K_{\varGamma }$ we let ${\varTheta }_k^m:= \{j : {\vec {q}}^m_k \in \overline{\sigma ^m_j}\}$ and set ${\varLambda }_k^m := \cup _{j \in {\varTheta }_k^m} \overline{\sigma ^m_j}$ and ${\vec {\omega }}^m_k := \frac{1}{\mathscr {H}^{d-1}({\varLambda }^m_k)} \sum _{j\in {\varTheta }_k^m} \mathscr {H}^{d-1}(\sigma ^m_j) \;{\vec {\nu }}^m_j$.

For the approximation to the velocity and pressure on ${\mathscr {T}}^m$ we use the finite element spaces $\mathbb {U}^m({\vec {g}})$ and $\mathbb {P}^m$, which are the direct time discrete analogues of $\mathbb {U}^h({\vec {g}})$ and $\mathbb {P}^h(t_m)$, as well as ${\widehat{\mathbb {P}}}^m \subset {\widehat{\mathbb {P}}}$. Analogously to (3.13), we also say that $(\mathbb {U}^m({\vec {0}}), \mathbb {P}^m,$ $W({\varGamma }^m), {\vec {\pi }}^m_\ell )$ satisfy the discrete LBB$_{\varGamma }$ inf-sup condition if there exists a $C_0 \in {\mathbb R}_{>0}$, independent of $\mathscr {T}^m$ and $\{\sigma ^m_j\}_{j=1}^{J_{\varGamma }}$, such that5.2where $\Vert \eta \Vert _{0,{\varGamma }^m}^2 := \left\langle \eta ,\eta \right\rangle _{{\varGamma }^m}$ and $\Vert {\vec {\eta }} \Vert _{1,{\varGamma }^m,h}^2 := \left\langle {\vec {\eta }},{\vec {\eta }} \right\rangle _{{\varGamma }^m} + \sum _{j = 1}^{J_{\varGamma }} \int _{\sigma _j^m} |\nabla _{\mathrm{s}}\,{\vec {\eta }}|^2 \;\mathrm{d}{\mathscr {H}}^{d-1}$. Unfortunately, it does not appear possible to prove that (5.2) holds for e.g. $(\mathbb {U}^m({\vec {0}}),$ $\mathbb {P}^m) = ([S^m_2({\varOmega })]^d \cap \mathbb {U}({\vec {0}}), S^m_1({\varOmega }))$, because $\mathscr {T}^m$ and ${\varGamma }^m$ are totally independent. Recall that also in the much simpler situation of the XFEM$_{\varGamma }$ approach from [8, 11], which corresponds to setting $\eta =0$ in (5.2) and replacing $\left\langle {\vec {\omega }}^m, {\vec {\xi }} \right\rangle _{{\varGamma }^m}^h$ with $\left\langle {\vec {\nu }}^m, {\vec {\xi }} \right\rangle _{{\varGamma }^m}$, the authors were unable to show that an LBB condition holds. In fact, in this simpler situation it is easily possible to construct a counterexample, e.g. when $\mathbb {P}^m = S^m_0({\varOmega })$. Then, if ${\varOmega }^m_-$ is a union of bulk elements, i.e. ${\varGamma }^m$ happens to be a union of bulk faces, clearly (5.2) does not hold, as $\mathrm {\mathscr {X}}_{{\varOmega }_-^m} \in \mathbb {P}^m$. Hence for the choice $\varphi = (\mathrm {\mathscr {X}}_{{\varOmega }_-^m} - \frac{\mathscr {L}^d({\varOmega }_-^m)}{\mathscr {L}^d({\varOmega })})$ and $\lambda = -1$ we obtain that

$$\begin{aligned} ( \varphi , \nabla \,.\,{\vec {\xi }}) + \lambda \left\langle {\vec {\nu }}^m, {\vec {\xi }} \right\rangle _{{\varGamma }^m} = 0 \qquad \forall \ {\vec {\xi }} \in \mathbb {U}^m({\vec {0}}). \end{aligned}$$

Of course, in practice this situation never occurs, because the totally independent ${\varGamma }^m$ is never exactly aligned with the bulk mesh.

Our proposed fully discrete equivalent of (4.19a, b), (4.9c–f), for a fixed $\ell \in \{1,2\}$, is then given as follows. Let ${\varGamma }^0$, an approximation to ${\varGamma }(0)$, as well as ${\vec {\kappa }}^0 \in \underline{V}({\varGamma }^0)$ and ${\vec {U}}^0\in \mathbb {U}^0({\vec {g}})$ be given. For $m=0,\ldots , M-1$, find ${\vec {U}}^{m+1} \in \mathbb {U}^m({\vec {g}})$, $P^{m+1} \in {\widehat{\mathbb {P}}}^m$, $P_\mathrm{sing}^{m+1} \in {\mathbb R}$, $P_{\varGamma }^{m+1} \in W({\varGamma }^m)$, ${\vec {X}}^{m+1}\in \underline{V}({\varGamma }^m)$, ${\vec {\kappa }}^{m+1}\in \underline{V}({\varGamma }^m)$ and ${\vec {F}}_{\varGamma }^{m+1} \in \underline{V}({\varGamma }^m)$ such that 5.3a
5.3b
5.3c
5.3d
5.3e
5.3f and set ${\varGamma }^{m+1} = {\vec {X}}^{m+1}({\varGamma }^m)$. Here we have defined ${\vec {f}}^{m+1} := {\vec {I}}^m_2\,{\vec {f}}(\cdot ,t_{m+1})$. We observe that (5.3a–f) is a linear scheme in that it leads to a linear system of equations for the unknowns $({\vec {U}}^{m+1}, P^{m+1}, P_\mathrm{sing}^{m+1}, P_{\varGamma }^{m+1}, {\vec {X}}^{m+1}, {\vec {\kappa }}^{m+1}, {\vec {F}}_{\varGamma }^{m+1})$ at each time level.

In the absence of the LBB$_{\varGamma }$ condition (5.2) we need to consider the reduced system (5.3a, d–f), where $\mathbb {U}^m({\vec {0}})$ in (5.3a) is replaced by $\mathbb {U}^m_0({\vec {0}})$. Here we define

5.4 $$\begin{aligned} \mathbb {U}^m_0({\vec {b}}) := \left\{ {\vec {U}} \in \mathbb {U}^m({\vec {b}}) : \right.&\left. (\nabla \,.\,{\vec {U}}, \varphi ) = 0 \ \ \quad \forall \ \varphi \in {\widehat{\mathbb {P}}}^m\,, \right. \nonumber \\&\left. \left\langle \nabla _{\mathrm{s}}\,.\,({\vec {\pi }}^m_\ell \,{\vec {U}}), \eta \right\rangle _{{\varGamma }^m} = 0 \ \ \quad \forall \ \eta \in W({\varGamma }^m)\right. \nonumber \\&\left. \text { and } \left\langle {\vec {U}}, {\vec {\omega }}^m \right\rangle _{{\varGamma }^m}^h = 0 \right\} , \end{aligned}$$

for given data ${\vec {b}} \in [C(\overline{{\varOmega }})]^d$.

In order to prove the existence of a unique solution to (5.3a–f) we make the following very mild well-posedness assumption.

$$(\mathscr {A})$$
We assume for $m=0,\ldots , M-1$ that $\mathscr {H}^{d-1}(\sigma ^m_j) > 0$ for all $j=1,\ldots , J_{\varGamma }$, and that ${\varGamma }^m \subset {\varOmega }$.

### **Theorem 5.1**

Let the assumption $(\mathscr {A})$ hold. If the LBB$_{\varGamma }$ condition (5.2) holds, then there exists a unique solution $({\vec {U}}^{m+1}, P^{m+1}, P_\mathrm{sing}^{m+1}, P_{\varGamma }^{m+1}, {\vec {X}}^{m+1}, {\vec {\kappa }}^{m+1}, {\vec {F}}_{\varGamma }^{m+1})$ $\in \mathbb {U}^m({\vec {g}})\times {\widehat{\mathbb {P}}}^m \times {\mathbb R}\times W({\varGamma }^m)\times [\underline{V}({\varGamma }^m)]^3$ to (5.3a–f). In all other cases, on assuming that $\mathbb {U}^m_0({\vec {g}})$ is nonempty, there exists a unique solution $({\vec {U}}^{m+1}, {\vec {X}}^{m+1}, {\vec {\kappa }}^{m+1}, {\vec {F}}_{\varGamma }^{m+1}) \in \mathbb {U}^m_0({\vec {g}}) \times [\underline{V}({\varGamma }^m)]^3$ to the reduced system (5.3a, d–f) with $\mathbb {U}^m({\vec {0}})$ replaced by $\mathbb {U}^m_0({\vec {0}})$.

### *Proof*

As the system (5.3a–f) is linear, existence follows from uniqueness. In order to establish the latter, we consider the homogeneous system. Find $({\vec {U}}, P, P_\mathrm{sing}, P_{\varGamma }, {\vec {X}}, {\vec {\kappa }},$ ${\vec {F}}_{\varGamma }) \in \mathbb {U}^m({\vec {0}})\times {\widehat{\mathbb {P}}}^m \times {\mathbb R}\times W({\varGamma }^m)\times [\underline{V}({\varGamma }^m)]^3$ such that 5.5a
5.5b
5.5c
5.5d
5.5e
5.5f Choosing ${\vec {\xi }}={\vec {U}}$ in (5.5a), $\varphi = P$ in (5.5b), $\eta = P_{\varGamma }$ in (5.5c), ${\vec {\chi }} = {\vec {F}}_{\varGamma }$ in (5.5d), ${\vec {\eta }}={\vec {\kappa }}$ in (5.5e) and ${\vec {\chi }} = {\vec {X}}$ in (5.5f) yields that5.6It immediately follows from (5.6), Korn’s inequality and $\alpha > 0$, that ${\vec {U}} = {\vec {0}} \in \mathbb {U}^m({\vec {0}})$ and ${\vec {\kappa }} = {\vec {0}}$. (For the application of Korn’s inequality we recall that $\mathscr {H}^{d-1}(\partial _1{\varOmega }) > 0$.) Hence (5.5d, f) yield that ${\vec {X}} = {\vec {0}}$ and ${\vec {F}}_{\varGamma }= {\vec {0}}$, respectively. Finally, if (5.2) holds then (5.5a) with ${\vec {U}} = {\vec {0}}$ and ${\vec {F}}_{\varGamma }= {\vec {0}}$ implies that $P = 0 \in {\widehat{\mathbb {P}}}^m$, $P_\mathrm{sing}= 0$ and $P_{\varGamma }= 0 \in W({\varGamma }^m)$. This shows existence and uniqueness of $({\vec {U}}^{m+1}, P^{m+1}, P_\mathrm{sing}^{m+1}, P_{\varGamma }^{m+1}, {\vec {X}}^{m+1}, {\vec {\kappa }}^{m+1},$ ${\vec {F}}_{\varGamma }^{m+1}) \in \mathbb {U}^m({\vec {g}})\times {\widehat{\mathbb {P}}}^m \times {\mathbb R}\times W({\varGamma }^m)\times [\underline{V}({\varGamma }^m)]^3$ to (5.3a–f). The proof for the reduced system is very similar. The homogeneous system to consider is (5.5a, d–f) with $\mathbb {U}^m({\vec {0}})$ replaced by $\mathbb {U}^m_0({\vec {0}})$. As before, we infer that (5.6) holds, which yields that ${\vec {U}} = {\vec {0}} \in \mathbb {U}^m_0({\vec {0}})$, ${\vec {\kappa }} = {\vec {0}}$, and hence ${\vec {X}} = {\vec {0}}$ and ${\vec {F}}_{\varGamma }= {\vec {0}}$. $\square$

### *Remark 5.1*

We always choose $\mathbb {U}^m({\vec {0}})$ and $\mathbb {P}^m$ so that the standard LBB condition in the bulk holds. Therefore $\mathbb {U}^m_0({\vec {g}})$, recall (5.4), is non-empty in the absence of the two ${\varGamma }^m$ constraints in (5.4). Clearly, there is the possibility of $\mathbb {U}^m_0({\vec {g}})$ being empty if the number of vertices on ${\varGamma }^m$, $K_{\varGamma }$, is too large compared to the number of bulk mesh vertices in the vicinity of ${\varGamma }^m$. In practice we refine our bulk meshes in the neighbourhood of the interface, which lessens the likelihood of this occurring. In fact, in practice we do not experience problems for our choices of bulk and surface meshes. For example, in the case $d=2$, we recall that the P2–(P1+P0) element satisfies the standard LBB condition in the bulk. This means that for the lowest order Taylor–Hood element P2–P1, which we employ in practice, there are some additional degrees of freedom in the velocity space, which prevent $\mathbb {U}^m_0({\vec {g}})$ from being empty in practice.

### *Remark 5.2*

The scheme (5.3a–f) clearly leads to a coupled system of linear equations. On replacing ${\vec {F}}_{\varGamma }^{m+1}$ in (5.3a) with ${\vec {F}}_{\varGamma }^m$ the system decouples into (5.3a–c) for $({\vec {U}}^{m+1}, P^{m+1}, P_\mathrm{sing}^{m+1}, P_{\varGamma }^{m+1})$ and into (5.3d–f) for $({\vec {X}}^{m+1}, {\vec {\kappa }}^{m+1}, {\vec {F}}_{\varGamma }^{m+1})$. Of course, the subsystem (5.3d–f) itself decouples into three equations for the three unknowns. While the decoupled system offers the advantage of being easier to solve, we found in practice that the coupled scheme (5.3a–f) preserved the surface area better than the decoupled scheme. An additional drawback of the decoupled scheme is that it is less stable and so in general needs smaller time steps than the coupled scheme (5.3a–f). The latter fact can partly be explained with the following observation.

On replacing ${\vec {F}}_{\varGamma }^{m+1}$ with ${\vec {\kappa }}^{m+1}$, and in the case $\partial _1{\varOmega }=\partial {\varOmega }$, ${\vec {g}} = {\vec {0}}$ and $\rho _{\varGamma }= 0$, we obtain an unconditionally stable approximation for two-phase flow in the spirit of [11], but with the additional side constraint (2.4c). In particular, for fixed bulk meshes in time one can show thatsee [11, Theorem 4.1] and [10, Theorem 4.2] for more details. It is for these reasons that we prefer the coupled scheme (5.3a–f).

## Solution methods

As is standard practice for the solution of linear systems arising from discretizations of Stokes and Navier–Stokes equations, we avoid the complications of the constrained pressure space ${\widehat{\mathbb {P}}}^m$ in practice by considering an overdetermined linear system with $\mathbb {P}^m$ instead. Introducing the obvious abuse of notation, the linear system (5.3a–c) for $\alpha =0$, with ${\widehat{\mathbb {P}}}^m$ replaced by $\mathbb {P}^m$, can be formulated as: Find $({\vec {U}}^{m+1},P^{m+1},P_\mathrm{sing}^{m+1},P_{\varGamma }^{m+1})$, such that

6.1 $$\begin{aligned} \begin{pmatrix} {\vec {B}}_{\varOmega }&{} {\vec {C}}_{\varOmega }&{} {\vec {D}}_{\varOmega }&{} {\vec {S}}_{{\varGamma },{\varOmega }}\\ {\vec {C}}^\mathrm{T}_{\varOmega }&{} 0 &{} 0 &{} 0 \\ {\vec {D}}^\mathrm{T}_{\varOmega }&{} 0 &{} 0 &{} 0 \\ {\vec {S}}_{{\varGamma },{\varOmega }}^T&{} 0 &{} 0 &{} 0 \end{pmatrix} \begin{pmatrix} {\vec {U}}^{m+1} \\ P^{m+1} \\ P_\mathrm{sing}^{m+1} \\ P_{\varGamma }^{m+1} \end{pmatrix} = \begin{pmatrix} {\vec {b}} \\ 0 \\ 0 \\ 0 \end{pmatrix}, \end{aligned}$$

where $({\vec {U}}^{m+1},P^{m+1},P_\mathrm{sing}^{m+1},P_{\varGamma }^{m+1})\in ({\mathbb R}^d)^{K^m_\mathbb {U}}\times {\mathbb R}^{K^m_\mathbb {P}} \times {\mathbb R}\times {\mathbb R}^{K_{\varGamma }}$ here denote the coefficients of these finite element functions with respect to the their standard bases. The definitions of the matrices and vectors in (6.1) directly follow from (5.3a–c), but we state them here for completeness in the case ${\vec {g}} = {\vec {0}}$. Let $i,\,j = 1 ,\ldots , K_\mathbb {U}^m$, $n,q = 1 ,\ldots , K_\mathbb {P}^m$ and $k,l = 1 ,\ldots , K_{\varGamma }$. Then6.2where $\{{\vec {e}}_r\}_{r=1}^d$ denotes the standard basis in ${\mathbb R}^d$, and where we have used the convention that the subscripts in the matrix notations refer to the test and trial domains, respectively. A single subscript is used where the two domains are the same. The entries of ${\vec {D}}_{\varOmega }$, for $i = 1 ,\ldots , K_\mathbb {U}^m$, are given by $[{\vec {D}}_{\varOmega }]_{i,1} := - \langle \phi _i^{\mathbb {U}^m}, {\vec {\omega }}^m \rangle _{{\varGamma }^m}^h$.

The only new term compared to previous works by the authors on two-phase flows, see [10, 11], is ${\vec {S}}_{{\varGamma },{\varOmega }}$. Here we note that

6.3 $$\begin{aligned} \left( \left\langle \chi ^{m}_l, \nabla _{\mathrm{s}}\,.\, (\pi ^m_\ell \,\phi _i^{\mathbb {U}^m}\,{\vec {e}}_r) \right\rangle _{\sigma ^m} \right) _{r=1}^d&= \left( \left\langle \chi ^{m}_l, \nabla _{\mathrm{s}}\,(\pi ^m_\ell \,\phi _i^{\mathbb {U}^m})\,.\,{\vec {e}}_r \right\rangle _{\sigma ^m} \right) _{r=1}^d. \end{aligned}$$

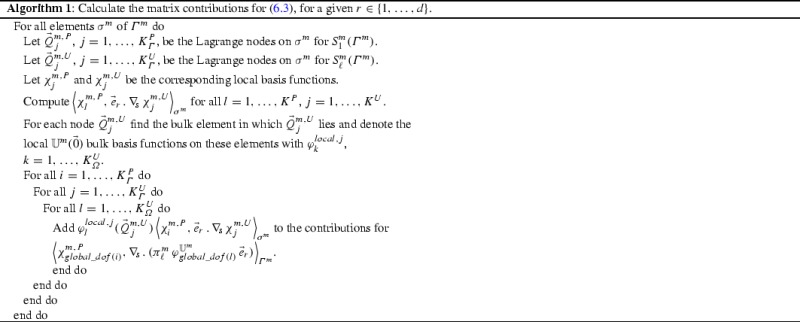


In order to provide a matrix-vector formulation for the full system (5.3a–f), and in particular in view of (5.3f), we recall from [18, p. 64] thatMoreover, we observe that $\langle \nabla _{\mathrm{s}}\,.\,{\vec {\xi }} , \nabla _{\mathrm{s}}\,.\,{\vec {\chi }} \rangle _{{\varGamma }^m} = \sum _{i,j=1}^d$ $\langle (\nabla _{\mathrm{s}})_j\,({\vec {\xi }})_j, (\nabla _{\mathrm{s}})_i\,({\vec {\chi }})_i \rangle _{{\varGamma }^m}$. Hence, in addition to (6.2), we introduce the following matrices, where $q = 1 ,\ldots , K_\mathbb {U}^m$, and $k,l = 1 ,\ldots , K_{\varGamma }$
Here we have made use of the fact that

$$\begin{aligned}{}[{\vec {\mathscr {B}}}_{\varGamma }]_{kl}&= \left( \left\langle \nabla _{\mathrm{s}}\,.\,(\chi ^m_l\,{\vec {e}}_j) , \nabla _{\mathrm{s}}\,.\,(\chi ^m_k\,{\vec {e}}_i) \right\rangle _{{\varGamma }^m}\right) _{i,j=1}^d = \left( \left\langle (\nabla _{\mathrm{s}}\,\chi ^m_l)\,.\,{\vec {e}}_j , (\nabla _{\mathrm{s}}\,\chi ^m_k)\,.\,{\vec {e}}_i \right\rangle _{{\varGamma }^m}\right) _{i,j=1}^d \nonumber \\&= \left( \left\langle [\nabla _{\mathrm{s}}]_j\,\chi ^m_l, [\nabla _{\mathrm{s}}]_i\,\chi ^m_k \right\rangle _{{\varGamma }^m} \right) _{i,j=1}^d. \end{aligned}$$

Moreover, it clearly holds that $([{\vec {\mathscr {B}}}_{\varGamma }]_{kl})^\mathrm{T} = [{\vec {\mathscr {B}}}_{\varGamma }]_{lk} =: [{\vec {\mathscr {B}}}^\star _{\varGamma }]_{kl}$.

Denoting the system matrix in (6.1) as $$\begin{pmatrix} {\vec {B}}_{\varOmega }&{} {\vec {\mathscr {C}}} \\ {\vec {\mathscr {C}}}^\mathrm{T} &{} 0 \end{pmatrix}$$, and letting $\widetilde{P}^{m+1} = (P^{m+1}, P_\mathrm{sing}^{m+1},$ $P_{\varGamma }^{m+1})^\mathrm{T}$, then the linear system (5.3a–f), with numerical integration in (5.3e, f), can be written as

6.4 $$\begin{aligned} \begin{pmatrix} {\vec {B}}_{\varOmega }&{} {\vec {\mathscr {C}}} &{} 0 &{} 0 &{} -\alpha \,{\vec {M}}_{{\varGamma },{\varOmega }}\\ \vec {\mathscr {C}}^\mathrm{T} &{} 0 &{} 0 &{} 0 &{} 0 \\ ({\vec {M}}_{{\varGamma },{\varOmega }})^T&{} 0 &{} 0 &{} -\frac{1}{\tau }\,{\vec {M}}_{\varGamma }&{} 0 \\ 0 &{} 0 &{} {\vec {M}}_{\varGamma }&{} {\vec {A}}_{\varGamma }&{} 0 \\ 0 &{} 0 &{} -{\vec {A}}_{\varGamma }&{} 0 &{} {\vec {M}}_{\varGamma }\end{pmatrix} \begin{pmatrix} {\vec {U}}^{m+1} \\ \widetilde{P}^{m+1} \\ {\vec {\kappa }}^{m+1} \\ \delta {\vec {X}}^{m+1} \\ {\vec {F}}^{m+1}_{\varGamma }\end{pmatrix} = \begin{pmatrix} {\vec {b}} \\ 0 \\ 0 \\ -{\vec {A}}_{\varGamma }\,{\vec {X}}^{m} \\ {\vec {\mathscr {Z}}}_{\varGamma }\,{\vec {\kappa }}^{m} + {\vec {A}}_{{\varGamma },{\vec {\kappa }}}\,{\vec {X}}^{m} \end{pmatrix}, \end{aligned}$$

where $\vec {\mathscr {Z}}_{\varGamma }:= {\vec {\mathscr {B}}}_{\varGamma }- {\vec {\mathscr {B}}}_{\varGamma }^\star - \vec {\mathscr {R}}_{\varGamma }$. For the solution of (6.4) a Schur complement approach similar to [11] can be used. In particular, the Schur approach for eliminating $({\vec {\kappa }}^{m+1},\delta {\vec {X}}^{m+1},{\vec {F}}^{m+1}_{\varGamma })$ from (6.4) can be obtained as follows. Let

$$\begin{aligned} {\varTheta }_{\varGamma }:= \begin{pmatrix} 0 &{} - \frac{1}{\tau }\,{\vec {M}}_{\varGamma }&{} 0\\ {\vec {M}}_{\varGamma }&{} {\vec {A}}_{\varGamma }&{} 0 \\ -{\vec {A}}_{\varGamma }&{} 0 &{} {\vec {M}}_{\varGamma }\end{pmatrix}. \end{aligned}$$

Then (6.4) can be reduced to

6.5a $$\begin{aligned}&\begin{pmatrix} {\vec {B}}_{\varOmega }+ \alpha \,{\vec {T}}_{\varOmega }&{} \vec {\mathscr {C}} \\ \vec {\mathscr {C}}^\mathrm{T} &{} 0 \end{pmatrix} \begin{pmatrix} {\vec {U}}^{m+1} \\ \widetilde{P}^{m+1} \end{pmatrix} = \begin{pmatrix} {\vec {b}} + \alpha \,{\vec {c}} \\ 0 \end{pmatrix} \end{aligned}$$

and

6.5b $$\begin{aligned} \begin{pmatrix} {\vec {\kappa }}^{m+1} \\ \delta {\vec {X}}^{m+1} \\ {\vec {F}}^{m+1}_{\varGamma }\end{pmatrix} = {\varTheta }_{\varGamma }^{-1}\, \begin{pmatrix} -({\vec {M}}_{{\varGamma },{\varOmega }})^T\,{\vec {U}}^{m+1} \\ -{\vec {A}}_{\varGamma }\,{\vec {X}}^{m} \\ {\vec {\mathscr {Z}}}_{\varGamma }\,{\vec {\kappa }}^{m} + {\vec {A}}_{{\varGamma },{\vec {\kappa }}}\,{\vec {X}}^m \end{pmatrix}. \end{aligned}$$

In (6.5a) we have used the definitions

$$\begin{aligned} {\vec {T}}_{\varOmega }= (0\ 0\ {\vec {M}}_{{\varGamma },{\varOmega }})\,{\varTheta }_{\varGamma }^{-1}\, {\begin{pmatrix} ({\vec {M}}_{{\varGamma },{\varOmega }})^T\\ 0 \\ 0 \end{pmatrix}} = \tau \,{\vec {M}}_{{\varGamma },{\varOmega }}\,{\vec {M}}_{\varGamma }^{-1}\,{\vec {A}}_{\varGamma }\,{\vec {M}}_{\varGamma }^{-1}\, {\vec {A}}_{\varGamma }\,{\vec {M}}_{\varGamma }^{-1}\,({\vec {M}}_{{\varGamma },{\varOmega }})^T\end{aligned}$$

and

$$\begin{aligned} {\vec {c}}&= (0\ 0\ {\vec {M}}_{{\varGamma },{\varOmega }})\,{\varTheta }_{\varGamma }^{-1}\, {\begin{pmatrix} 0 \\ - {\vec {A}}_{\varGamma }\,{\vec {X}}^{m} \\ {\vec {\mathscr {Z}}}_{\varGamma }\,{\vec {\kappa }}^{m} + {\vec {A}}_{{\varGamma },{\vec {\kappa }}}\,{\vec {X}}^m \end{pmatrix}} \\&= {\vec {M}}_{{\varGamma },{\varOmega }}\,{\vec {M}}_{\varGamma }^{-1}\,[{\vec {\mathscr {Z}}}_{\varGamma }\,{\vec {\kappa }}^m + {\vec {A}}_{{\varGamma },{\vec {\kappa }}}\,{\vec {X}}^m - {\vec {A}}_{\varGamma }\,{\vec {M}}_{\varGamma }^{-1}\,{\vec {A}}_{\varGamma }\,{\vec {X}}^m]. \end{aligned}$$

For the linear system (6.5a) well-known solution methods for finite element discretizations for the standard Navier–Stokes equations may be employed. We refer to [11, § 5], where we describe such solution methods in detail for a very similar situation.

## Numerical results

For the bulk mesh adaptation we use the strategy from [11], which results in a fine mesh size $h_f$ around ${\varGamma }^m$ and a coarse mesh size $h_c$ further away from it. Here $h_{f} = \frac{2\,\min \{H_1,H_2\}}{N_{f}}$ and $h_{c} = \frac{2\,\min \{H_1,H_2\}}{N_{c}}$ are given by two integer numbers $N_f > N_c$, where we assume from now on that the convex hull of ${\varOmega }$ is given by $\times _{i=1}^d (-H_i,H_i)$. To summarize the discretization parameters we use the shorthand notation $n\,\mathrm{adapt}_{k,l}$ from [11]. The subscripts refer to the fineness of the spatial discretizations, i.e. for the set $n\,\mathrm{adapt}_{k, l}$ it holds that $N_f = 2^k$ and $N_c = 2^l$. For the case $d=2$, in this paper, we have in addition that $K_{\varGamma }= J_{\varGamma }= 2^k + 1$. Finally, the uniform time step size for the set $n\,\mathrm{adapt}_{k,l}$ is given by $\tau = 10^{-3} / n$, and if $n=1$ we write $\mathrm{adapt}_{k, l}$. We remark that we implemented the scheme (5.3a–f) with the help of the finite element toolbox ALBERTA, see [37].

In all the numerical simulations we employ the scheme with numerical integration in (5.3e, f), i.e. we choose the superscript $\cdot ^h$ in the two brackets $\cdot ^{(h)}$. Moreover, unless otherwise stated, we use the scheme (5.3a–f) with $\ell = 1$. The initial data ${\vec {\kappa }}^0 \in \underline{V}({\varGamma }^0)$ is always computed as the solution to

$$\begin{aligned} \left\langle {\vec {\kappa }}^{0} , {\vec {\eta }} \right\rangle _{{\varGamma }^0}^{h} + \left\langle \nabla _{\mathrm{s}}\,{\vec {\mathrm{id}}}, \nabla _{\mathrm{s}}\,{\vec {\eta }} \right\rangle _{{\varGamma }^0} = 0 \qquad \forall \ {\vec {\eta }} \in \underline{V}({\varGamma }^0). \end{aligned}$$

In addition, we employ the lowest order Taylor–Hood element P2–P1 in all computations and set ${\vec {U}}^0 = {\vec {I}}^0_2\,{\vec {u}}_0$. Unless stated otherwise we fix $\partial _1{\varOmega }= \partial {\varOmega }$, ${\vec {g}} = {\vec {0}}$ and ${\vec {u}}_0 = {\vec {0}}$. The volume force is always set to ${\vec {f}} = {\vec {0}}$. Moreover, we set all physical parameters to unity, i.e. $\rho _\pm = \mu _\pm = \rho _{\varGamma }= \mu _{\varGamma }= \alpha = 1$, unless stated otherwise.

At times we will discuss the discrete energy of the numerical solutions. On recalling Theorem 4.1 the discrete energy is defined by

$$\begin{aligned} \mathscr {E}^h({\varGamma }^m, {\vec {\kappa }}^{m+1}) := \mathscr {E}_{kin}^h({\varGamma }^m, \rho ^m , {\vec {U}}^{m+1}) + \tfrac{1}{2}\,\alpha \left\langle {\vec {\kappa }}^{m+1}, {\vec {\kappa }}^{m+1}\right\rangle _{{\varGamma }^m}^{h}, \end{aligned}$$

where

$$\begin{aligned} \mathscr {E}_{kin}^h({\varGamma }^m, \rho ^m , {\vec {U}}^{m+1}) := \tfrac{1}{2}\, \Vert [\rho ^m]^\frac{1}{2}\,{\vec {U}}^{m+1} \Vert _0^2 + \tfrac{1}{2}\, \rho _{\varGamma }\left\langle {\vec {U}}^{m+1}, {\vec {U}}^{m+1} \right\rangle _{{\varGamma }^m}^h \end{aligned}$$

represents the kinetic part of the discrete energy. For the simulation of vesicles the reduced volume is often mentioned as a characteristic number. In the case $d=3$, and for the initial discrete interface ${\varGamma }^0$, this is defined as

$$\begin{aligned} v_r = \frac{3\,\mathscr {L}^3({\varOmega }^0_-)}{4\,\pi \,(\frac{\mathscr {H}^2({\varGamma }^0)}{4\,\pi })^\frac{3}{2}} = \frac{6\,\pi ^\frac{1}{2}\,\mathscr {L}^3({\varOmega }^0_-)}{({\mathscr {H}^2({\varGamma }^0))^\frac{3}{2}}}, \end{aligned}$$

see e.g. [42]. In a similar fashion, for the case $d=2$ we define the reduced area as

$$\begin{aligned} a_r = \frac{\mathscr {L}^2({\varOmega }^0_-)}{\pi \,(\frac{\mathscr {H}^1({\varGamma }^0)}{2\,\pi })^2} = \frac{4\,\pi \,\mathscr {L}^2({\varOmega }^0_-)}{({\mathscr {H}^1({\varGamma }^0))^2}}. \end{aligned}$$

### Numerical simulations in 2D

For all our two-dimensional simulations we choose the discretization parameters $2\,\mathrm{adapt}_{9,4}$. In all the simulations presented here the areas of the two phases, as well as the length of the interface, are well preserved, with the relative differences over time in each case being less than $0.2\,\%$. Moreover, the ratio

$$\begin{aligned} r_a:=\max _{j\in \{1,\ldots , J_{\varGamma }\}} \mathscr {H}^{d-1} (\sigma ^m_j) / \min _{j\in \{1,\ldots , J_{\varGamma }\}} \mathscr {H}^{d-1} (\sigma ^m_j) \end{aligned}$$

of the largest and smallest elements’ lengths was always bounded by 1.005. Here we note that we always choose the initial polygon ${\varGamma }^0$ to be equidistributed.

We conducted the following shearing experiments on the domain ${\varOmega }= (-2,2)^2$ for an initial interface in the form of an ellipse, centred at the origin, with axis lengths 1 and 2.5, so that $a_r = 0.745$. In particular, we prescribe the inhomogeneous Dirichlet boundary condition ${\vec {g}}({\vec {z}}) = (z_2, 0)^\mathrm{T}$ on $\partial _1{\varOmega }= [-2,2] \times \{\pm 2\}$. For the initial data ${\vec {u}}_0$ we choose the function ${\vec {u}}_0({\vec {z}}) = \eta (z_2)\,{\vec {e}}_1$, where $\eta : [-2,2] \rightarrow {\mathbb R}$ is a continuous piecewise linear function with $\eta (\pm 2) = \pm 2$ and $\eta (s) = 0$ if $|s| \le 1.5$. Hence ${\vec {u}}_0$ satisfies the required conditions $\nabla \,.\,{\vec {u}}_0 = 0$ in ${\varOmega }$ and $\nabla _{\mathrm{s}}\,.\,{\vec {u}}_0 = 0$ on ${\varGamma }(0)$, recall (2.8), and is such that ${\vec {u}}_0 = {\vec {g}}$ on $\partial _1{\varOmega }$. The remaining parameters are given by $\alpha = 0.05$, $\rho _\pm = \rho _{\varGamma }= \mu _{\varGamma }= 1$ and either

**Fig. 2:**
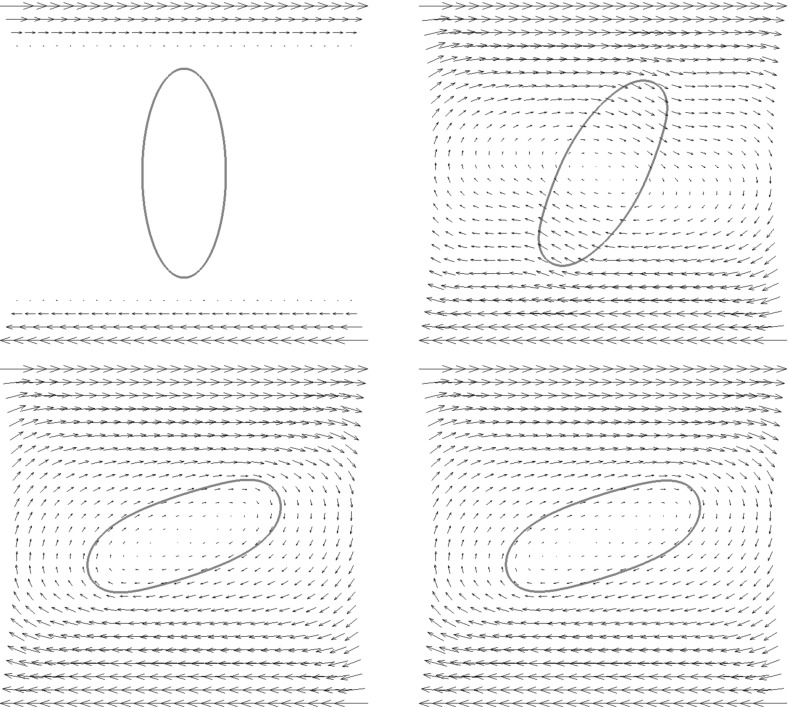
Shear flow with parameters as in (7.1a), leading to tank treading. *The plots* show the interface ${\varGamma }^m$, together with the discrete velocity ${\vec {U}}^m$ on $\overline{{\varOmega }}$, at times $t=0,\ 1,\ 3,\ 5$ (*top left to bottom right*)

**Fig. 3:**
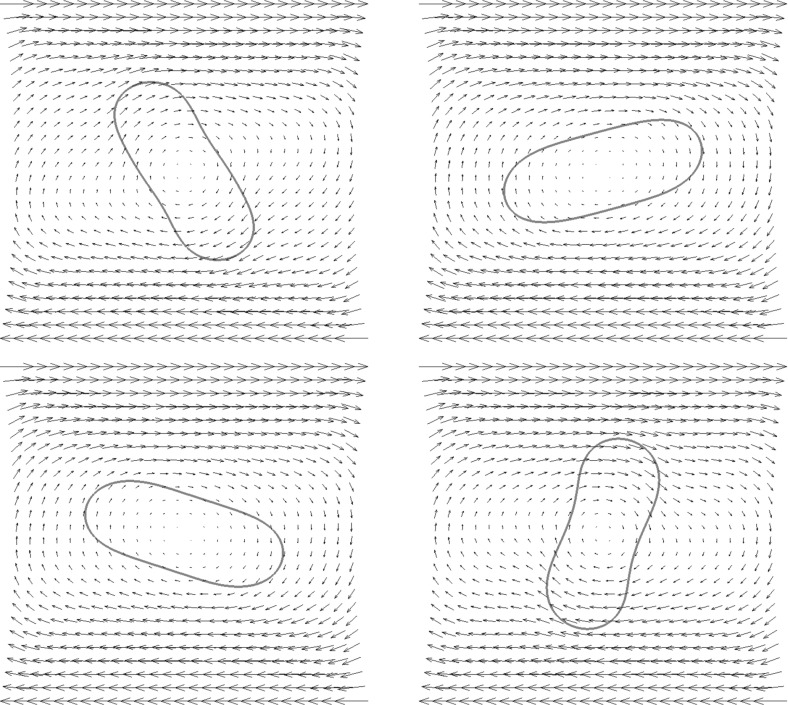
Shear flow with parameters as in (7.1b), leading to tumbling. *The plots* show the interface ${\varGamma }^m$, together with the discrete velocity ${\vec {U}}^m$ on $\overline{{\varOmega }}$, at times $t=8,\ 11,\ 14,\ 17$ (*top left to bottom right*)

7.1 $$\begin{aligned} \text {(a)}\quad \mu _+ = 1,\quad \mu _- = 1\,,\quad \text {or}\quad \text {(b)}\quad \mu _+ = 1,\quad \mu _- = 10, \end{aligned}$$

where we recall that the continuous problem, as $d=2$, is independent of the value of $\mu _{\varGamma }$. The results can be seen in Figs. 2 and 3. In the first case we observe that the evolution reaches a steady state in which the interfacial fluid rotates along the interface. This motion is often called tank treading, see e.g. [36]. The second example, on the other hand, leads to a rotation of the whole vesicle, and this is called tumbling.

**Fig. 4:**
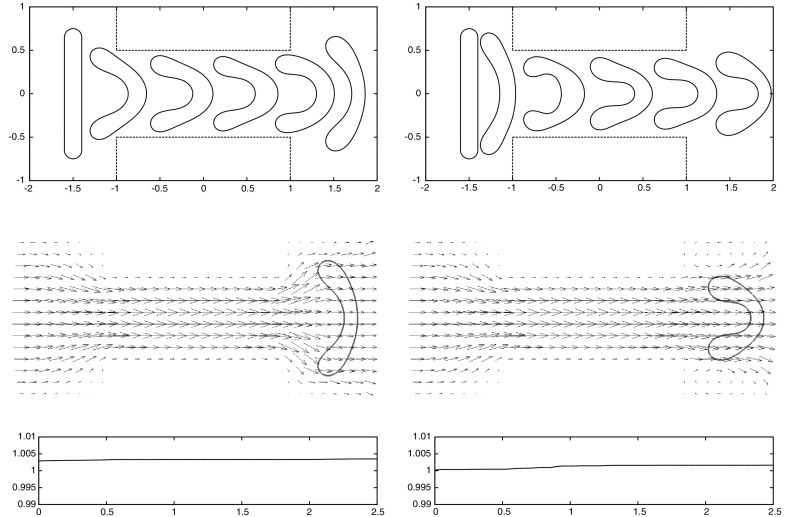
Flow through a constriction for the scheme (5.3a–f). *Left* for $\rho _{\varGamma }= 0$ and *right* for $\rho _{\varGamma }= 15$, where the *plots* show the interface ${\varGamma }^m$ at times $t=0,\ 0.5,\ 1,\ 1.5,\ 2,\ 2.5$. The *middle row* shows a visualization of ${\vec {U}}^{m}$ at time $t=2.5$, and below are plots of the ratio $r_a$

The numerical simulation of a vesicle flowing through a constriction can be seen in Fig. 4. This example shows that membranes can drastically deform in order to pass through a constriction. This resembles the remarkable properties of red blood cells, which show a similar behaviour when flowing through capillaries. Here we choose the initial shape of the interface to be an elongated tube of total dimension $0.2\times 1.5$. This gives a reduced area of $a_r = 0.351$. As the computational domain we choose ${\varOmega }= (-2,2) \times (-1,1) \setminus ( [-1,1]\times [-1,-0.5] \cup [-1,1]\times [0.5,1] )$ with $\partial _2{\varOmega }= \{2\} \times (-1,1)$ and $\partial _1{\varOmega }= \partial {\varOmega }\setminus \partial _2{\varOmega }$. On the left boundary $\{-2\} \times [-1,1]$ we prescribe the inhomogeneous boundary conditions ${\vec {g}}({\vec {z}}) = (1 - z_2^2, 0)^\mathrm{T}$ in order to model Poiseuille flow. In this computation we consider the quasi-static variant with $\rho _\pm = 0$. Moreover, we set $\alpha = 0.1$ and let $\rho _{\varGamma }= 0$ or $\rho _{\varGamma }=15$. In the latter case the effect of inertia on the evolution is clearly visible. We note that for the experiment with $\rho _{\varGamma }=0$, the ratio $r_a$ increases at the very first time step from 1 to 1.0023, which explains the graph in Fig. 4. Here we recall from Remark 4.1 that equidistribution is only maintained for the semidiscrete variant of our scheme.

**Fig. 5:**
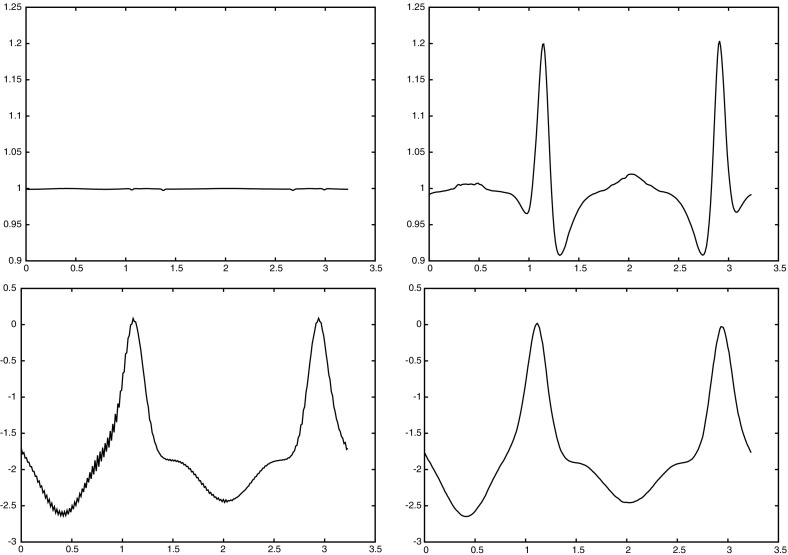
The final distribution of the scalar field ${\varPsi }^M\in W({\varGamma }^{M})$, plotted against arclength, for the simulation in Fig. 4 with $\rho _{\varGamma }= 0$. On the *left* for $\ell = 1$, on the *right* for $\ell = 2$. The *second row* visualizes the surface pressures $P_{\varGamma }^M \in W({\varGamma }^{M-1})$, plotted against arclength

**Fig. 6:**
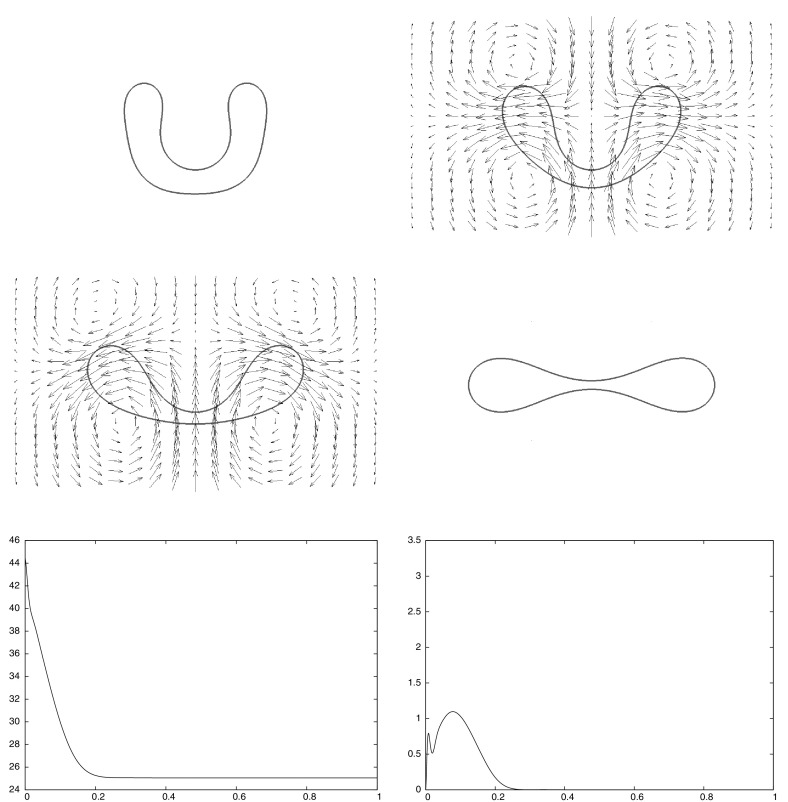
Flow for a smooth letter “C” for $\rho _{\varGamma }= 0$. The *plots* show the interface ${\varGamma }^m$, together with the discrete velocity ${\vec {U}}^m$ on $[-1,1]\times [-0.5,0.5]$, at times $t=0,\ 0.05,\ 0.1,\ 1$. *Below are plots* of the discrete energy and the discrete kinetic energy

**Fig. 7:**
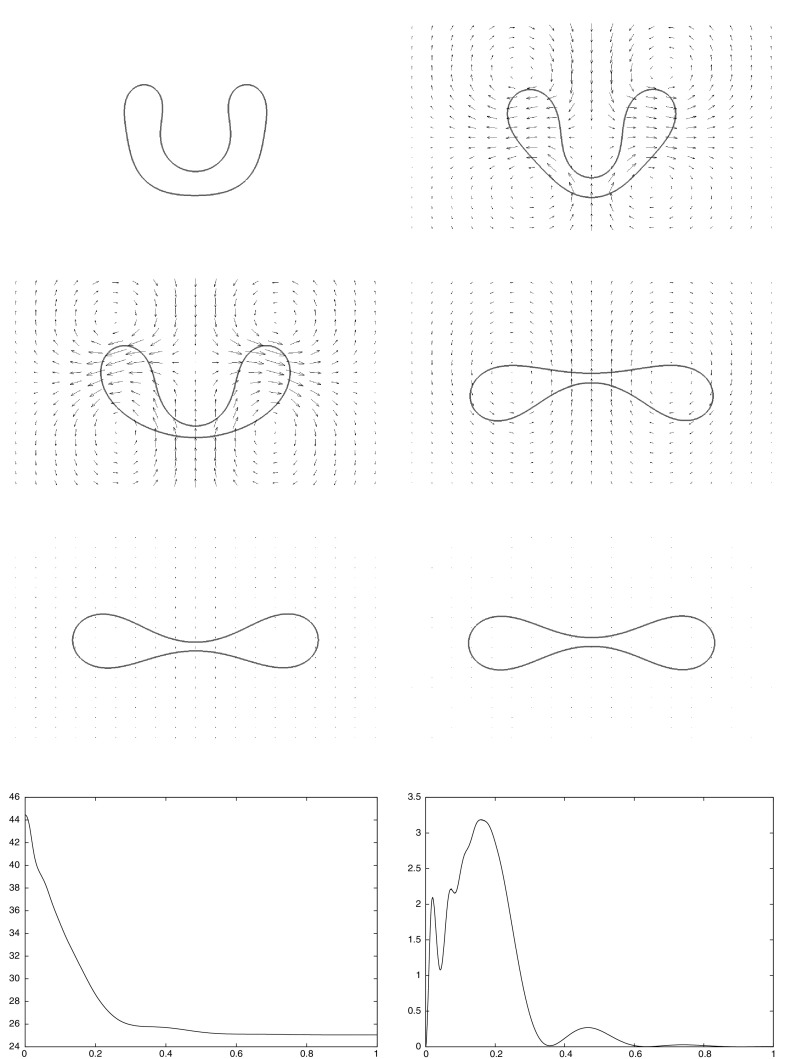
Flow for a smooth letter “C” for $\rho _{\varGamma }= 1$. The *plots* show the interface ${\varGamma }^m$, together with the discrete velocity ${\vec {U}}^m$ on $[-1,1]\times [-0.5,0.5]$, at times $t=0,\ 0.05,\ 0.1,\ 0.3,\ 0.6,\ 1$. *Below are plots* of the discrete energy and the discrete kinetic energy

**Fig. 8:**
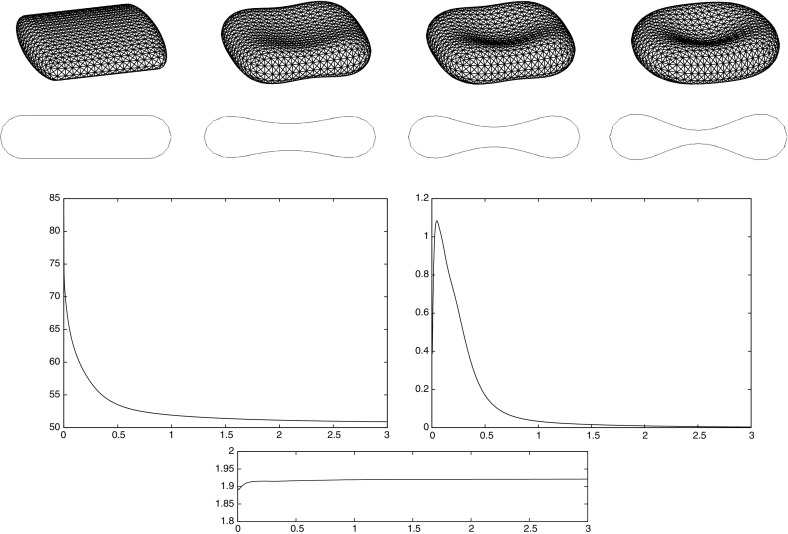
Flow for a flat plate of dimensions $4\times 4\times 1$ for the scheme (5.3a–f). The triangulations of ${\varGamma }^m$ at times $t=0,\ 0.5,\ 1,\ 3$, together with cuts of ${\varGamma }^m$ at $z_2 = 0$. The *third row* shows plots of the discrete energy and the discrete kinetic energy, while a plot of the ratio $r_a$ can be found at the *bottom*

**Fig. 9:**
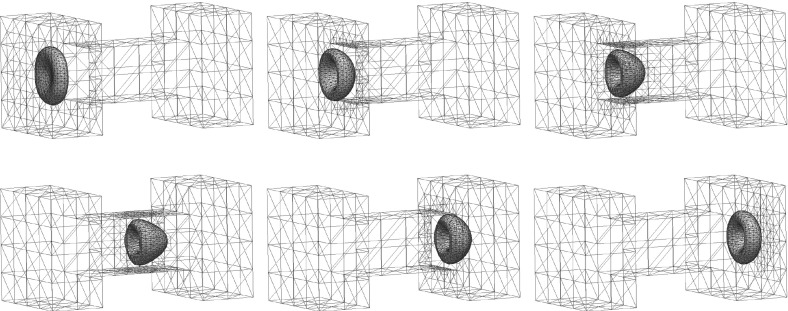
Flow through a constriction. The *plots* show the interface ${\varGamma }^m$ at times $t=0,\ 0.3,\ 0.5,\ 1,\ 1.4,\ 1.8$

**Fig. 10:**
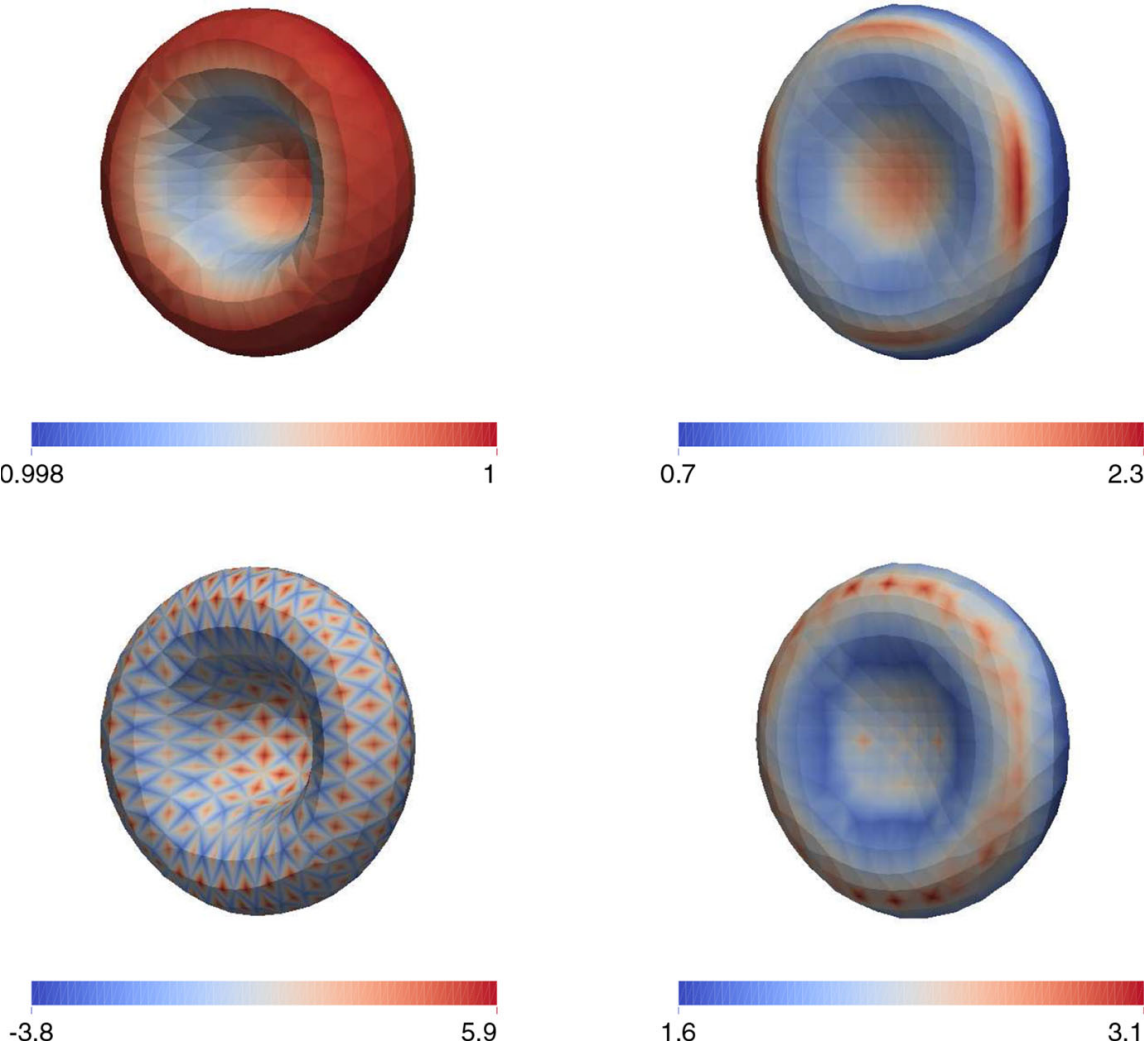
The *first row* shows the final distribution of the scalar field ${\varPsi }^M\in W({\varGamma }^{M})$, while the *second row* shows the final surface pressure $P_{\varGamma }^{M} \in W({\varGamma }^{M-1})$. *On the left* for $\ell = 1$, and *on the right* for $\ell = 2$

**Fig. 11:**
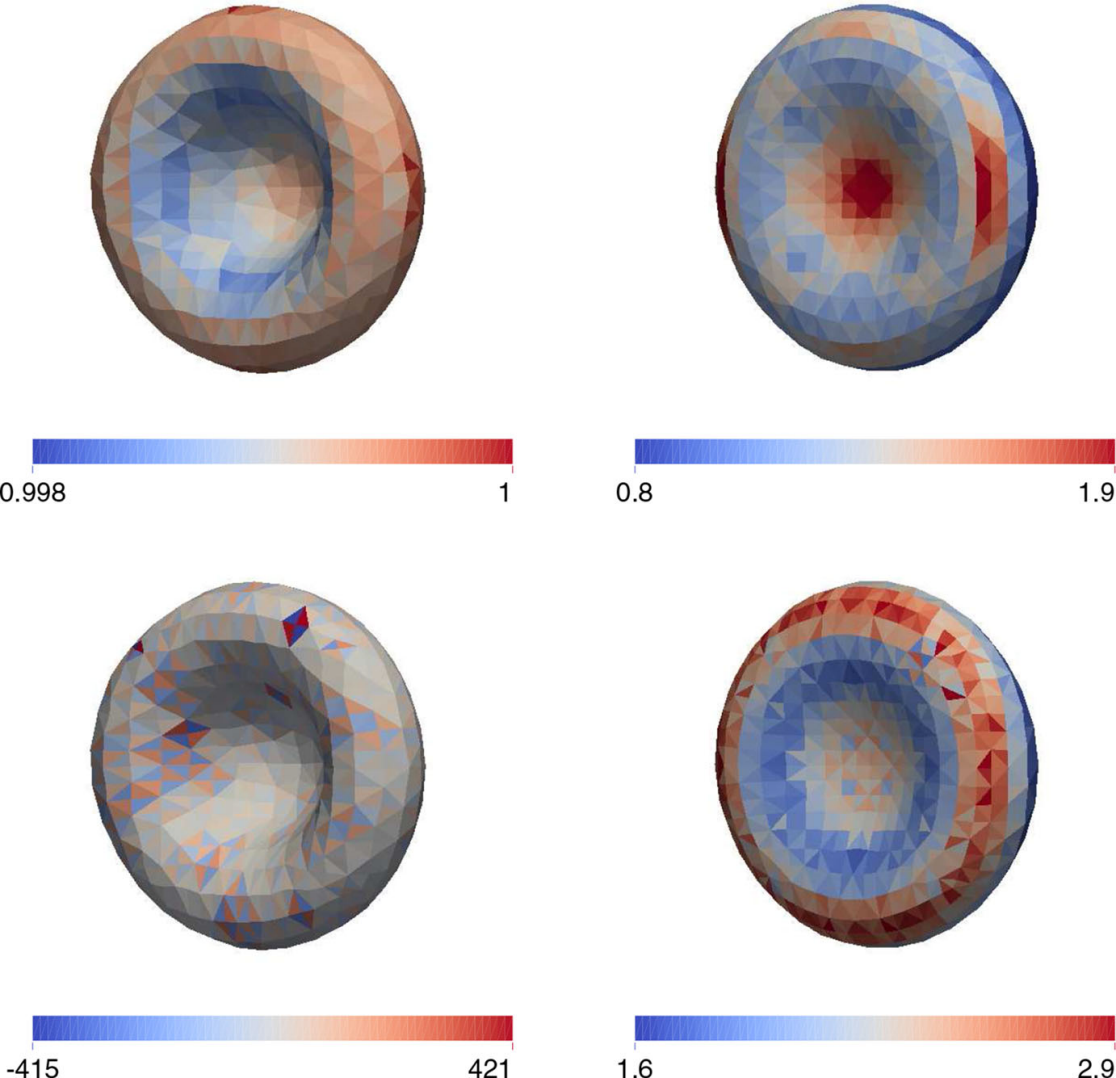
The *first row* shows the final distribution of the scalar field scalar field ${\varPsi }^M\in S^M_0({\varGamma }^{M})$, while the *second row* shows the final surface pressure $P_{\varGamma }^{M} \in S^{M-1}_0({\varGamma }^{M-1})$. *On the left* for $\ell = 1$, and *on the right* for $\ell = 2$

**Fig. 12:**
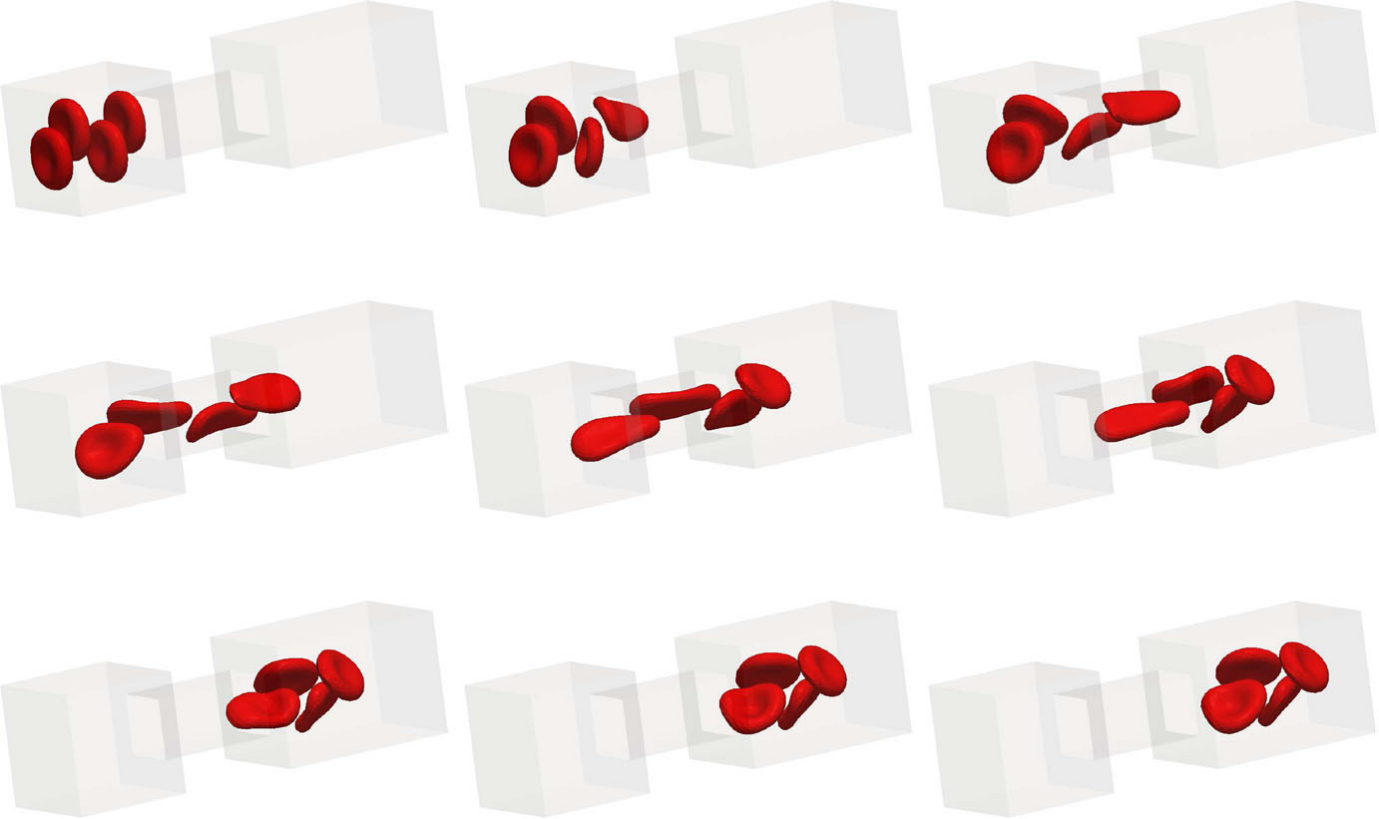
Flow through a constriction. The *plots* show the interface ${\varGamma }^m$ at times $t=0,\ 0.5,\ 1,\ 1.5,\ 2,\ 2.5,\ 3,\ 3.5,\ 4$

In order to highlight the *local* interface length preservation, we also show in Fig. 5 how a scalar field, initialized as ${\varPsi }^0 = 1$, is transported along the interface by the fluid. To this end, at each time step, we find ${\varPsi }^{m+1} \in W({\varGamma }^{m+1})$ such that

7.2 $$\begin{aligned} \left\langle {\varPsi }^{m+1}, \chi ^{m+1}_k \right\rangle _{{\varGamma }^{m+1}}^h = \left\langle {\varPsi }^{m}, \chi ^{m}_k \right\rangle _{{\varGamma }^m}^h\qquad \forall \ k \in \{1,\ldots ,K_{\varGamma }\}, \end{aligned}$$

recall (4.4e) in [9] without diffusion. We compare the results to a simulation for the scheme (5.3a–f) with $\ell = 2$, when no local interface length preservation can be expected, recall Remark 4.2. Clearly, using the scheme with $\ell = 2$ leads to a nonuniform distribution of the scalar field ${\varPsi }^M$, which coincides with a nonuniform distribution of the vertices along the discrete interface ${\varGamma }^M$. Despite this difference in the approximation of ${\varGamma }(t_M)$, the two simulations produce interfaces ${\varGamma }^M$ that are nearly identical. This suggests that the oscillatory surface pressure exhibited by the scheme (5.3a–f) with $\ell = 1$ compared to $\ell = 2$, see the bottom row in Fig. 5, does not have a detrimental effect on the velocity approximation.

A very pronounced difference between $\rho _{\varGamma }= 0$ and $\rho _{\varGamma }> 0$ can be observed in our next simulation, where we start with an initial shape in the form of a smooth letter “C” with reduced area $a_r = 0.326$. As the computational domain we choose ${\varOmega }= (-1,1)^2$, and we let $\rho _\pm = \alpha = 1$. See Figs. 6 and 7 for the evolutions in the cases $\rho _{\varGamma }= 0$ and $\rho _{\varGamma }= 1$, respectively. In the latter case, the two arms of the vesicle swing up and down due to inertia, which is clearly visible in the plot of the kinetic energy as well.

### Numerical simulations in 3D

Unless otherwise stated, for the uniform time step size we choose $\tau =10^{-3}$ in this subsection. In all the simulations presented here the volumes of the two phases, as well as the area of the interface, are almost exactly preserved, with the relative differences over time in each case being less than $0.2\,\%$.

As a first example for a three-dimensional simulation, we consider the evolution for an initially flat plate of total dimension $4\times 4\times 1$, similarly to [6, Fig. 15]. As the computational domain we choose ${\varOmega }= (-2.5,2.5)^3$, and we set $\rho _{\varGamma }= 0$. We note that the reduced volume for this shape is given by $v_r = 0.569$. As discretization parameters we choose adapt$_{5,2}$, and the initial triangulation ${\varGamma }^0$ satisfies $(K_{\varGamma },J_{\varGamma }) = (1538, 3072)$ and $r_a = 1.898$. The results for the scheme (5.3a–f) can be seen in Fig. 8, where we note that the interface assumes the shape of a red blood cell. Plots of the discrete energies and of the ratio $r_a$ are also shown in Fig. 8. We note that the discrete energy is monotonically decreasing, while the ratio $r_a$ always remains bounded below 2.

The numerical simulation of a vesicle flowing through a constriction can be seen in Fig. 9. Here we choose the initial shape of the interface to be a biconcave surface resembling a human red blood cell, with a reduced volume of $v_r = 0.568$. As the computational domain we choose ${\varOmega }= (-2,-1) \times (-1,1)^2 \cup [-1,1]\times (-0.5,0.5)^2 \cup (1,2) \times (-1,1)^2$. We define $\partial _2{\varOmega }= \{2\} \times (-1,1)^2$ and on $\partial _1{\varOmega }$ we set no-slip conditions, except on the left hand part $\{-2\} \times [-1,1]^2$, where we prescribe the inhomogeneous boundary conditions ${\vec {g}}({\vec {z}}) = ([1 - z_2^2 - z_3^2]_+, 0, 0)^\mathrm{T}$ in order to model a Poiseuille-type flow. For the remaining parameters we set $\rho = \rho _{\varGamma }= 0$, $\mu = 1$ and $\alpha = 0.1$. and $\alpha =0.1$. As discretization parameters we choose adapt$_{5,2}$, and the initial triangulation ${\varGamma }^0$ satisfies $(K_{\varGamma },J_{\varGamma }) = (770, 1536)$.

Similarly to in Fig. 5, we consider the transport of a scalar field ${\varPsi }^0 = 1$ on ${\varGamma }^0$ for the simulation in Fig. 9. As can be seen from the plot in Fig. 10, the local surface area is maintained almost exactly throughout the evolution. The same cannot be said for the scheme (5.3a–f) with $\ell = 2$. Here the final distribution of ${\varPsi }^M$ is very uneven, and the overall surface area decreases by $14\,\%$. As a consequence, the final shape ${\varGamma }^M$ differs dramatically from the run with $\ell = 1$.

We also show the final surface pressure $P_{\varGamma }^{M}$ in Fig. 10, and also compare the results with a run for the scheme (5.3a–f) with $\ell = 2$. Similarly to the results in Fig. 5, we observe oscillatory surface pressures when $\ell = 1$, while $\ell = 2$ yields well behaved surface pressure approximations. The same simulation for piecewise constant surface pressures $P^{m+1} \in S^m_0({\varGamma }^m)$, and for both $\ell = 1$ and $\ell = 2$, yields the plots in Fig. 11. Here we replace (7.2) with the suitable formulation for the basis functions of $S^m_0({\varGamma }^m)$ and $S^{m+1}_0({\varGamma }^{m+1})$. We observe that this evolution can be computed also for piecewise constant surface pressure approximations. However, for more complex evolutions, such as three dimensional analogues of Figs. 2 and 3, we observe locking. Here the iterative linear solver is unable to find a discrete solution, even at the first time step.

Finally, in the larger domain ${\varOmega }= (-3,-1) \times (-1,1)^2 \cup [-1,1]\times (-0.5,0.5)^2 \cup (1,4) \times (-1,1)^2$, with the analogous boundary conditions, we also show the flow of four vesicles through a constriction, see Fig. 12. Here we choose adapt$_{4,1}$ for our original scheme (5.3a–f) with $\ell = 1$, and the initial triangulation ${\varGamma }^0$ satisfies $(K _{\varGamma },J_{\varGamma }) = (4\times 770, 4\times 1536)$.
